# KLF2/PPARγ axis contributes to trauma‐induced heterotopic ossification by regulating mitochondrial dysfunction

**DOI:** 10.1111/cpr.13521

**Published:** 2023-06-21

**Authors:** Ziyang Sun, Hang Liu, Yuehao Hu, Gang Luo, Zhengqiang Yuan, Weixuan Liu, Bing Tu, Hongjiang Ruan, Juehong Li, Cunyi Fan

**Affiliations:** ^1^ Department of Orthopedics Shanghai Sixth People's Hospital Affiliated to Shanghai Jiao Tong University School of Medicine Shanghai China; ^2^ Shanghai Engineering Research Center for Orthopaedic Material Innovation and Tissue Regeneration Shanghai China; ^3^ Shanghai Key Laboratory of Orthopaedic Implants, Department of Orthopaedic Surgery, Shanghai Ninth People's Hospital Shanghai Jiao Tong University School of Medicine Shanghai China

## Abstract

Trauma‐induced heterotopic ossification (HO) is a complex disorder after musculoskeletal injury and characterized by aberrant extraskeletal bone formation. Recent studies shed light on critical role of dysregulated osteogenic differentiation in aberrant bone formation. Krupel‐like factor 2 (KLF2) and peroxisome proliferator‐activated receptor gamma (PPARγ) are master adapter proteins that link cellular responses to osteogenesis; however, their roles and relationships in HO remain elusive. Using a murine burn/tenotomy model in vivo, we identified elevated KLF2 and reduced PPARγ levels in tendon stem/progenitor cells (TSPCs) during trauma‐induced HO formation. Both KLF2 inhibition and PPARγ promotion reduced mature HO, whereas the effects of PPARγ promotion were abolished by KLF2 overexpression. Additionally, mitochondrial dysfunction and reactive oxygen species (ROS) production also increased after burn/tenotomy, and improvements in mitochondrial function (ROS scavenger) could alleviate HO formation, but were abolished by KLF2 activation and PPARγ suppression by affecting redox balance. Furthermore, in vitro, we found increased KLF2 and decreased PPARγ levels in osteogenically induced TSPCs. Both KLF2 inhibition and PPARγ promotion relieved osteogenesis by improving mitochondrial function and maintaining redox balance, and effects of PPARγ promotion were abolished by KLF2 overexpression. Our findings suggest that KLF2/PPARγ axis exerts regulatory effects on trauma‐induced HO through modulation of mitochondrial dysfunction and ROS production in TSPCs by affecting redox balance. Targeting KLF2/PPARγ axis and mitochondrial dysfunction can represent attractive approaches to therapeutic intervention in trauma‐induced HO.

## Graphical abstract

Summary of effects and mechanisms of KLF2/PPARγ pathway mediated mitochondrial dysfunction that regulate traumatic HO progression. After burn/tenotomy, tendon stem/progenitor cells (TSPCs) accumulate in the tendon lesions, and the elevated expression of KLF2 further promotes PPARγ dependent mitochondrial dysfunction and ROS production by affecting redox balance in the TSPCs, resulting in enhanced expression of osteogenic genes and inducing osteogenesis differentiation, and finally leads to HO formation. Further, all of AAVs downregulate KLF2, PPARγ promotor Rosiglitazone, and ROS scavenger XJB‐5‐131 could suppress mitochondrial dysfunction involved in these biological processes, thereby inhibiting the HO formation.

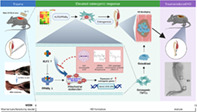

## 1 BACKGROUND

Acquired or traumatic heterotopic ossification (HO) is a condition that leads to the formation of mature lamellar bone in extraskeletal soft tissues, triggered by combat‐related injuries, fractures and dislocations, deep burns and central nervous system injuries.^1^ HO causes joint dysfunction, which seriously affects the quality of life and imposes a heavy health and economic burden on families and society. Non‐steroidal anti‐inflammatory drugs and radiation therapy are the main treatment methods for the prevention of HO.^2^, ^3^ However, the incidence of post‐traumatic HO remains high, with reports of up to 40%.^4^, ^5^ Thus, it is important to fully understand the complex mechanism of HO formation and explore effective methods for prevention.

There is increasing evidence suggesting that stem cells are capable of self‐renewal and multidirectional differentiation to repair damaged tissues.^6^ Ideally, when a tendon injury occurs, stem cells accumulate in damaged tissue and differentiate to generate repair. However, in a local inflammatory microenvironment, some stem cells undergo osteogenesis, which is an important pathological basis for HO formation.^7^ Previous studies have shown that tendon stem/progenitor cells (TSPCs), typically marked by platelet‐derived growth factor receptor alpha (PDGFRα), play a key role in the formation of chondrocytes and osteocytes during HO development.^8^ However, the mechanisms underlying TSPCs osteogenic differentiation at the time of HO formation remain unclear.

Krupel‐like factor (KLF) is a member of the zinc finger transcription factor family and 17 members of the mammalian family have been identified. KLF2 is an important member of the KLF family and plays an important regulatory role in many cellular biological processes,^9^, ^10^ including osteogenesis.^11^ Previous studies have shown that KLF2 stimulates osteogenic differentiation of stem cells from the periodontal ligament and promotes the regeneration of damaged teeth.^12^ However, to our knowledge, the role of KLF2 in HO formation remains unclear.

Peroxisome proliferator‐activated receptor gamma (PPARγ) is a member of the nuclear hormone receptor superfamily of ligand‐responsive transcription factors. PPARγ is an important cellular and metabolic switch that mediates several physiological and disease processes.^13^ Previous studies have shown that PPARγ is a metabolic switch that determines the fate of bone marrow mesenchymal stem cells (BMSCs), and its activation promotes adipogenic differentiation, whereas its inhibition promotes osteogenic differentiation.^14^, ^15^ Furthermore, PPARγ has also been reported to regulate the osteogenic response to some extent via KLF2.^16^ To date, no studies have investigated whether PPARγ influences the formation of HO.

Mitochondria are the main sources of cellular energy and play an irreplaceable role in a wide range of biological cellular processes. Previous studies have reported that mitochondrial dysfunction promotes osteogenic differentiation of BMSCs.^17^ Additionally, a recent Biosign report revealed 3547 differentially expressed proteins during HO formation, which were primarily enriched in oxidative phosphorylation and regulation of mitochondrial function.^18^ This implies that mitochondria may play an overwhelming regulatory role in HO formation. Furthermore, recent studies have found that PPARγ inhibition impairs mitochondrial function, indicating a close relationship between PPARγ and mitochondrial function.^19^

The objective of this study was to identify the role of the KLF2/PPARγ axis in traumatic HO and to reveal the potential contribution of mitochondrial dysfunction as the effector mechanism of the KLF2/PPARγ axis in the formation of HO.

## 2 METHODS

### 2.1 Reagents and materials

Alpha‐minimum essential medium (α‐MEM) medium and fetal bovine serum (FBS) were purchased from Gibco. The penicillin/streptomycin were purchased from HyClone. Rosiglitazone (Cat #S2556) and GW9662 (Cat #S2915) were purchased from Selleck Chemicals. XJB‐5‐131 (Cat #HY‐129460) was purchased from MedChemExpress. Adeno‐associated virus (AAV) and plasmids were purchased from Genomeditech Co., Ltd. The anti‐Runx2 (Cat #20700), OPN (Cat #22951), OCN (Cat #23418), PPARγ (Cat #16643) and SOD2 (Cat #24127) antibodies were purchased from Proteintech. The anti‐PDGFRα (Cat #AF1062) and CAT (Cat #AF3398) antibodies were purchased from R&D Systems. Anti‐KLF2 (Cat #A16480) antibody was purchased from Abclone. The GAPDH (Cat #GB1100C2) and β‐actin (Cat #GB15001) antibodies were obtained from Servicebio. The anti‐SREBP1 (Cat #28481) antibody was obtained from Abcam Biotechnology.

### 2.2 Establishment of the murine burn/tenotomy model (trauma‐induced HO) murine model, specimen collection and histological observations

C57BL/6 male mice aged 8–10 weeks were raised under special pathogen‐free conditions at the Animal Experimental Center of the Shanghai Sixth People's Hospital. Mice had free access to food and water and were acclimatized to the environment for at least 1 week before modelling. All experimental protocols for animals were approved by the Institutional Animal Care and Use Committee of Shanghai Sixth People's Hospital (IACUC no. DWLL2023‐0398), and the experimental procedures were conducted in accordance with the National Institutes of Health Guidelines for the Care and Use of Laboratory Animals.

In this study, a murine burn/tenotomy model was used to mimic trauma‐induced HO referring to Peterson.^20^ Mice were anesthetized with 1% pentobarbital sodium and a longitudinal incision of approximately 0.5 cm was made on the medial aspect of the bilateral distal hindlimbs. The Achilles tendon was exposed and transected at its midpoint, followed by closure of the skin incision with a 5‐0 vicryl stitch. Then an aluminium block of 2 cm × 2 cm × 3 cm, weighing 35 g and covering approximately 30% of the body area surface, was preheated to 60°C in a water bath. After the tenotomy, the block was placed on the exposed shaved dorsal skin and maintained for 17 s. Sham surgery was performed only by exposing the Achilles tendon without tenotomy or burn injury.

Mice were sacrificed at different time points for histological observation at 3 and 10 weeks to detect chondro‐osteogenesis and mature HO, respectively. Specifically, the harvested skin of the lower extremities was carefully removed and soft tissue was acquired from the musculotendinous junction to the calcaneus enthesis. The tissues were fixed in 10% (v/v) formalin. After 10 weeks, an additional decalcification step was performed using a 19% ethylenediaminetetraacetic acid solution. The decalcified tissues were dehydrated and embedded in paraffin. The tissue sections were obtained longitudinally along the Achilles tendon at a thickness of 5 μm. The stained sections on Thermo Superfrost®Plus slides were further deparaffinized and rehydrated for staining preparation. High‐throughput whole transcriptome sequencing, routine histological (Safranin O and Fast Green [SOFG] staining, haematoxylin and eosin [H&E] staining), immunohistochemistry (IHC) and immunofluorescence (IF) staining, micro‐computed tomography (micro‐CT), quantitative real‐time reverse transcriptase polymerase chain reaction (qPCR) and western blot (WB) were carried out to analyse the changes in tissue morphology and composition.

### 2.3 High‐throughput whole transcriptome sequencing

Whole transcript genome sequencing was performed on the soft tissue at the Achilles tendon (provided by Shanghai OE Biotech. Co., Ltd). The steps are as follows: total RNA was extracted and ribosomal RNA was digested using ribo‐Zero kit. After interrupt reagent was added to break the RNA into short fragments, a chain was synthesized with random primer of six bases using the interrupted RNA as template cDNA and then prepared a two‐strand synthesis reaction system to synthesize two‐strand cDNA, dUTP instead of dTTP in the synthesis of cDNA and then one strand containing dUTP was digested by UNG enzyme method, and only the cDNAs of the different junctions of the strand were retained. A chain used kit to purify a strand of cDNA, a strand of purified cDNA was then repaired at the end, an a‐tail was added and sequenced. Then the fragment size was selected and PCR was performed. The constructed libraries were qualified by Agilent 2100 Bioanalyzer. Illumina sequencer was used for sequencing.

### 2.4 IHC and IF staining

After the sections were prepared, citrate buffer was used for thermal remediation to retrieve antigens. The sections were then inactivated using hydrogen peroxide and blocked with goat serum. Sections were then incubated overnight with primary antibodies against KLF2 (1:200), PPARγ (1:200), PDGFRα (1:1000) and RUNX2 (1:100). On the second day, for IHC staining, sections were incubated with horseradish peroxidase (HRP)‐conjugated secondary antibody (1:500). For IF staining, sections were incubated at 37°C for 1 h using Alexa Fluor 488 conjugated secondary antibody or cy3 matching species (1:500) and 4,6‐diamidino‐2‐phenylindole (DAPI, Cat #P0131, Beyotime Institute of Biotechnology) was used for nuclear counterstaining.

### 2.5 Quantitative real‐time reverse transcriptase polymerase chain reaction

Relative expression levels of KLF2, PPARγ, SOD2 and CAT mRNA were detected using qPCR, with GAPDH as an internal control. Total RNA was isolated from tissues using TRIzol reagent (Invitrogen) according to the manufacturer's instructions and using reverse transcriptase was converted to complementary DNA using M‐MLV reverse transcriptase (Takara). Target gene expression was quantified using SYBR Green Premix Ex Taq (Takara Bio). The qPCR data were exported and processed using the 2^−ΔΔCt^ method. The primer sequences are listed in Table S1.

### 2.6 Western blotting

Tissues (in vivo) or whole cells (in vitro) from different groups were collected and lysed with radioimmunoprecipitation assay lysis buffer (Servicebio) containing a protease inhibitor cocktail (Servicebio). The lysate was sonicated on ice and the entire tissue protein was extracted at 12,000 RPM for 10 min. Protein concentrations were determined using a bisphenol acid protein detection kit (Servicebio). Equal amounts of protein (30 μg) were suspended in sodium dodecyl sulfate‐polyacrylamide electrophoresis gel loading buffer (SDS‐PAGE) (Servicebio), heated at 100°C for 5 min and separated by electrophoresis on 12% SDS‐PAGE. The proteins were then transferred onto a polyvinylidene difluoride membrane. After blocking the membrane with 5% non‐fat milk or bovine serum albumin for 1 h at room temperature, it was incubated with the corresponding primary antibodies (KLF2, PPARγ, RUNX2, OCN, OPN, TFAM, PGC1α, SREBP1, SOD2 and CAT) and against glyceraldehyde 3‐phosphate dehydrogenase (GAPDH) overnight at 4°C. After incubation with HRP‐conjugated secondary antibodies for 1 h at room temperature, an enhanced chemiluminescence reagent was applied to form a signal, which was detected using a ChemiDoc CRS imaging system (Bio‐Rad). The corresponding relative density of the grey protein bands was calculated using ImageJ software (version 1.32, National Institutes of Health). Briefly, the integrated optical density (IntDen) of each protein band of interest was determined. The relative grey level was obtained by normalizing the IntDen of each protein of interest to that of the internal reference protein GAPDH.

### 2.7 Micro‐CT scanning

At 10 weeks, the mice were sacrificed and the hindlimbs of each group were collected and fixed with 10% formalin (v/v) for 48 h. The acquired hindlimbs were subsequently scanned using a Skyscan 1176 high‐resolution micro‐CT scanner (software version 1.1 [build 6], Bruker). The isometric resolution was 18 mm and the voltage was 70 kV. The CTvox software (version) was used to obtain 3D reconstructed images. Bone volume was calculated using CTan software (Version 1.15.4.0+, Bruker), and a high‐density mass in soft tissue with Hounsfield units greater than 272 was considered HO.

### 2.8 Reactive oxygen species staining

The frozen sections were re‐warmed at room temperature and dried. The sections were then placed in phosphate‐buffered saline (PBS; pH = 7.4) and washed three times with shaking on a decolourization shaker for 5 min each. The nuclei were stained with DAPI staining solution and incubated for 10 min in the dark. The slides were washed and sealed with an antifluorescence quenching sealer. Finally, images were acquired using a fluorescent microscope.

### 2.9 Cell culture and osteogenic induction

TSPCs were isolated from the Achilles tendon of 4‐ to 6‐week‐old C57BL/6 mice as previously described.^21^ The tendon tissue was minced and digested with type I collagenase (Sigma‐Aldrich) on a shaker at 37°C for 2 h. Cell suspensions were cultured in a basic medium (α‐MEM, 10% FBS, 100 U/mL penicillin and 100 μg/mL streptomycin [all from Gibco]) in an incubator at 37°C and 5% CO_2_. The medium was changed every 3 days to maintain adequate nutritional conditions for the cells. The fully grown cells were trypsinized, spread in 6‐well or 24‐well plates, and incubated with osteo‐inductive medium (containing 10% serum, 50 μg/mL vitamin C, 10 mmol/L β‐glycerophosphate disodium salt and dexamethasone 0.1 μmol/L) when the cells reached an abundance of 80%–90% in the well plates; different treatments were applied to the cells depending on the different groups. qPCR, WB analysis, cell IF staining, alkaline phosphatase (ALP) staining, Alizarin red S (ARS) staining, JC1 staining and mitotracker staining were carried out to analyse the changes in cell morphology and composition.

### 2.10 Cell IF staining

After treatment, cells were fixed in 4% paraformaldehyde and permeabilized with 0.5% Triton X‐100. Cells were blocked with BSA (1%) and incubated at 4°C overnight with anti‐KLF2 (1:100), anti‐PPARγ (1:200), anti‐RUNX2 (1:200), anti‐OCN (1:200), anti‐OPN (1:500), anti‐SREBP (1:200), anti‐SOD2 (1:100), anti‐CAT (1:50) and anti‐TOMM20 (1:500) antibodies. Cells were stained with conjugated secondary antibodies in the dark. The nuclei were counterstained with DAPI and the cells were scanned using a digital slide scanner (Pannoramic MIDI; 3DHISTECH Ltd.).

### 2.11 JC1 staining

After treating the TSPCs under different conditions and performing osteogenic culture, the cells were washed three times with PBS and then incubated with JC1 dye for 20 min at 37°C. After washing again with PBS, the cells were mounted on glass slides and viewed under a fluorescent microscope (Olympus Corporation of the Americas, Slide book 5.0 × 64 software ix81).

### 2.12 Mitotracker staining

Petri plates were covered with an appropriate amount of medium to cover the coverslips for climbing culture. When cells had grown to 70% abundance, the culture medium was aspirated and the working solution for MitoTrackerRed/Green FM staining was added before washing at 37°C. Incubate for 15–45 min under normal culture conditions for the cells used (optimal incubation time). After staining, the staining solution was replaced with fresh culture medium or buffer and observed under a fluorescent microscope.

### 2.13 ALP staining

TSPCs were washed three times with pre‐cooled PBS and lysed on ice in pre‐cooled 1% Triton X‐100 for 30 min. Cell lysate ALP activity was determined and its value at 405 nm was normalized to the total protein concentration. TSPCs were washed three times with PBS, fixed with 4% paraformaldehyde for 10 min and incubated with NBT‐BCIP solution (Beyotime Biotech Company) for another 15 min. The images were captured under a microscope (Olympus DP73 Microscope; Olympus). ALP activity was measured using an ALP assay kit (Nanjing Jiancheng Biotechnology Co., Ltd.) according to the manufacturer's instructions.

### 2.14 ARS staining

The osteogenesis of TSPCs was induced for 14 days. The cells were washed, fixed in 95% ethanol for 14 min and stained with 2% ARS‐Tris‐HCL solution (pH 4.3). Mineralized nodules were observed using an inverted microscope. ARS staining was eluted with 10% cetylpyridine chloride and absorbance was measured at 570 nm using a SpectraMax i3x (Molecular Devices).

### 2.15 Oil Red O staining

Adipogenic differentiation medium A (Induction medium) and medium B (Maintenance medium; Cyagen) were prepared according to product instructions. The cells were inoculated in 24‐well plates and induced when the cell density reached 80%. About 2 mL of medium A was added to each well for 3 days, and replaced by medium B for 24 h. Then change the medium back to medium A. After 3 weeks of incubation, TSPCs were washed with PBS and fixed with 10% formalin for 20 min. After washing the cells two times with PBS, Oil red O staining was performed to observe the extent of lipid drop accumulation respectively.

### 2.16 Statistical analysis

Data analysis was performed using GraphPad Prism 9. Data are expressed as mean ± standard deviation (SD). Data were checked for normality using the Shapiro–Wilk test, and homogeneity of variance was determined using one‐way analysis of variance (ANOVA). Comparisons between groups were performed using one‐way ANOVA followed by Tukey's test for post hoc comparisons when the data were normally distributed; otherwise, Kruskal–Wallis tests for non‐normally distributed data. Categorical data were compared using the chi‐square test. Statistical significance was established at *p* < 0.05, and two‐tailed tests were performed.

## 3 RESULTS

### 3.1 The expression of KLF2 was elevated in tendon lesions in burn/tenotomy mice

**FIGURE 1:**
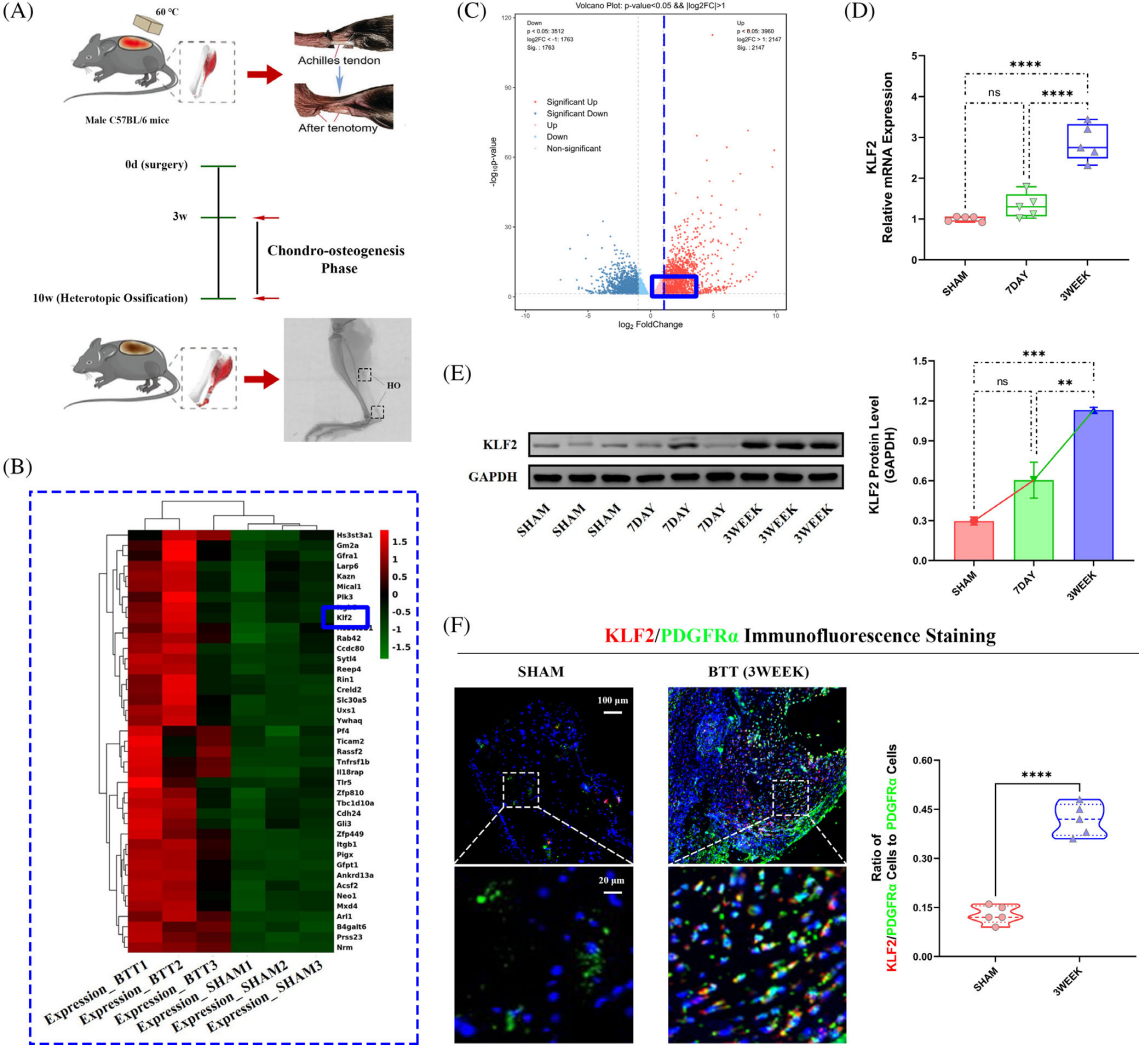
Increased expression of KLF2 in vivo after burn/tenotomy. (A) Schematic depiction of the establishment of a murine burn/tenotomy model and different stages of heterotopic ossification progression. The mice were randomly divided into three groups: (i) SHAM (sham surgery) and (ii‐iii) BTT (burn/tenotomy, sacrificed at 7 days [7DAY] and 3 weeks [3WEEK] after surgery) groups. (B, C) High‐throughput whole‐transcriptome sequencing was performed and showed by heat map and volcano plot in the tendon lesions at 3 weeks. *N* = 3. (D) qPCR was used to detect the relative mRNA levels (normalized to GAPDH) of KLF2 from the tendon lesions at indicated days. *N* = 5; ****p < 0.0001, ns = no significance. (E) WB analysis was used to detect the expression of KLF2 in the tendon lesions at indicated days. *N* = 3; **p < 0.01, ***p < 0.001, ns = no significance. (F) IF staining was used to detect the positive cells of KLF2 (red), co‐localized with PDGFR‐α (green), in the tendon lesions at 3 weeks. *N* = 5; scale bar = 100 μm (original magnification) and 20 μm (insert magnification of the boxed area, 5.0×); ****p < 0.0001. IF, immunofluorescence; qPCR, quantitative real‐time reverse transcriptase polymerase chain reaction; WB, western blot.

A mouse burn/tenotomy model was established according to the literature (Figure 1A). To explore the underlying mechanism of HO, high‐throughput whole transcriptome sequencing was performed on tendon lesions after 3 weeks, and the data were uploaded into the NCBI repository (bioproject no. GSE233201). Between the two groups (i) SHAM and (ii) BTT, both the heat map (Figure 1B) and the volcano map (Figure 1C) indicated a significantly elevated KLF2 expression in BTT group. Further, qRT‐PCR (KLF2; Figure 1D), WB analysis (KLF2; Figure 1E) and IF staining (KLF2, co‐localized with PDGFRα, a marker for the detection of tendon stem cells, Figure 1F) also indicated increased KLF2 expression in BTT group at 3 weeks.

### 3.2 KLF2 promoted trauma‐induced HO formation in burn/tenotomy mice

**FIGURE 2:**
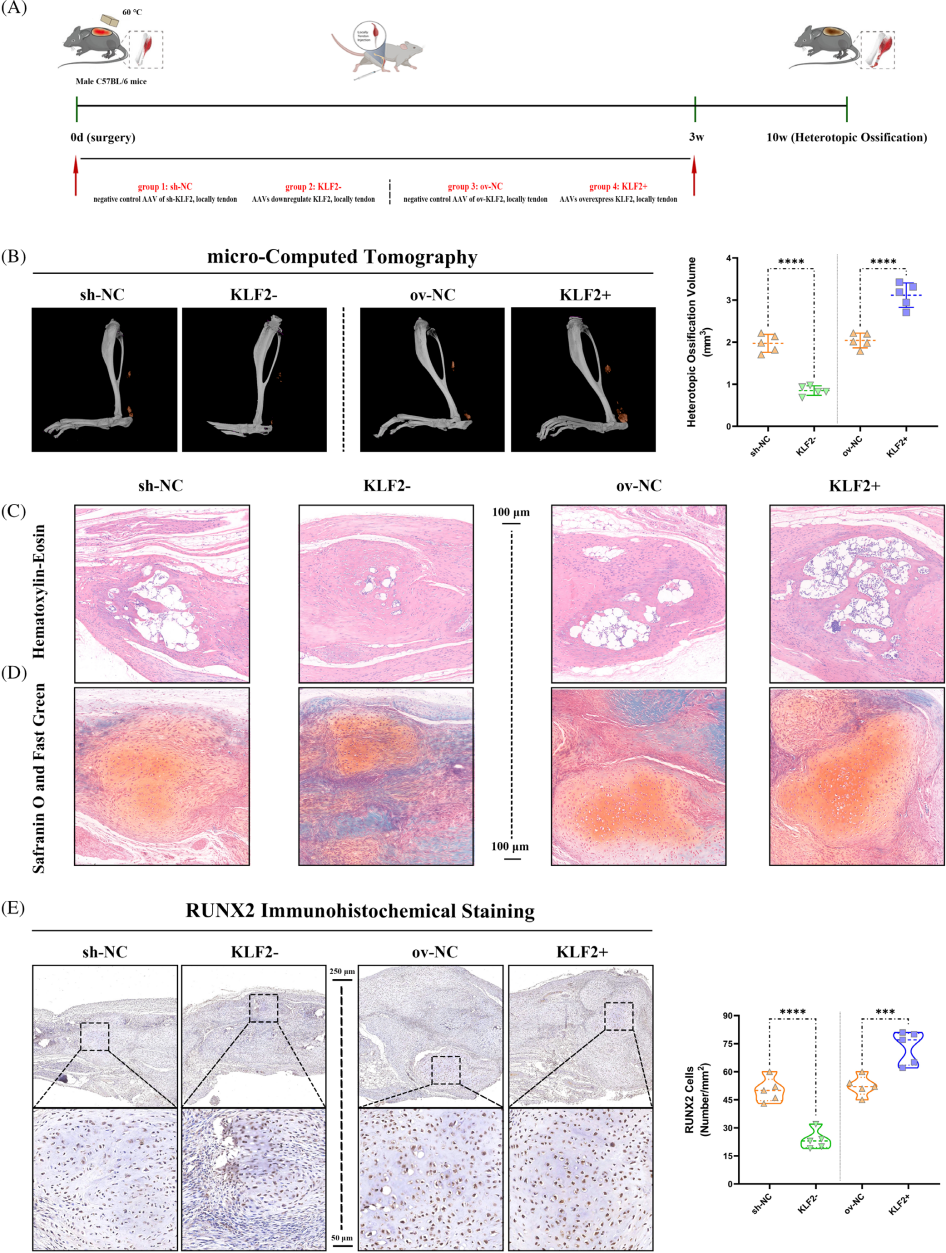
Reduced trauma‐induced HO formation after KLF2 inhibition in vivo after burn/tenotomy. (A) Schematic depiction of the intervention protocols for animal experiments. The mice were randomly assigned into four groups: (i) sh‐NC (burn/tenotomy with locally tendon downregulation vehicle [AAV harbouring no downregulation sequence] injection), (ii) KLF2− (burn/tenotomy with locally tendon sh‐KLF2 [AAV downregulate KLF2] injection), (iii) ov‐NC (burn/tenotomy with locally tendon overexpression vehicle [AAV harbouring no overexpression sequence] injection) and (iv) KLF2+ (burn/tenotomy with locally tendon ov‐KLF2 [AAV overexpress KLF2] injection). (B, C) Micro‐CT (B) and H&E staining (C) were used to detect HO formation at 10 weeks in the tendon lesions. The volume of HO was quantified from each group. *N* = 5; scale bar = 100 μm; ****p < 0.0001. (D) SOFG staining was used to detect chondrogenesis at 3 weeks in the tendon lesions. *N* = 5; scale bar = 100 μm. (E) IHC staining was used to detect osteogenesis by the positive cells of RUNX2 in the tendon lesions at 3 weeks. *N* = 5; scale bar = 250 μm (original magnification) and 50 μm (insert magnification of the boxed area, 5.0×); ****p < 0.0001, ***p < 0.001. H&E, haematoxylin & eosin; HO, heterotopic ossification; IHC, immunohistochemistry; micro‐CT, micro‐computed tomography.

Now that confirming that elevated levels of KLF2 were present in the chondro‐osteogenesis stage, to determine the effects of KLF2 on trauma‐induced HO formation in vivo, the mature HO and chondro‐osteogenesis indicators were evaluated between burn/tenotomy mice and the same model administrated with AAVs down‐regulated or overexpressed KLF2 (Figure 2A). Between the two groups (i) sh‐NC, and (ii) KLF2−, micro‐CT (Figure 2B), H&E staining (Figure 2C), SOFG staining (Figure 2D) and IHC staining (RUNX2; Figure 2E) all confirmed that KLF2− group had markedly decreased matured HO at 10 weeks and chondro‐osteogenesis at 3 weeks post‐tendon injury. However, between the two groups (i) ov‐NC, and (ii) KLF2+, opposite effects were observed (Figure 2B–E). The above results indicated that KLF2 deficiency attenuated trauma‐induced HO formation in burn/tenotomy mice.

### 3.3 PPARγ participated in the regulation of KLF2 in trauma‐induced HO formation in burn/tenotomy mice

Taking into account the central role of PPARγ in osteogenesis supported by recent studies, the potential relationship between KLF2 and PPARγ in the formation of HO was further investigated.

**FIGURE 3:**
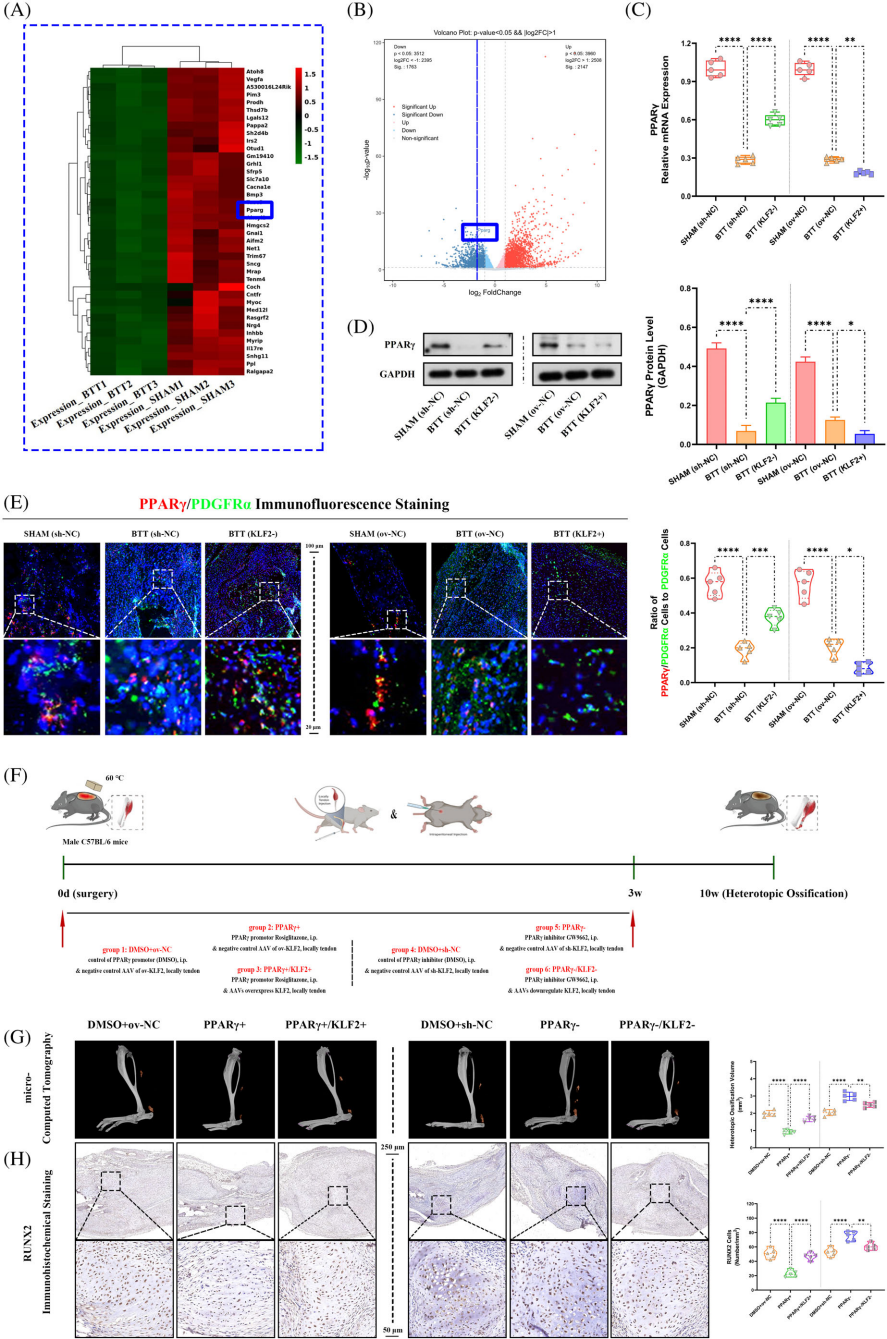
Role of PPARγ in regulation of KLF2 on trauma‐induced HO formation in burn/tenotomy mice. (A, B) High‐throughput whole‐transcriptome sequencing was performed and showed by heat map and volcano plot in the tendon lesions at 3 weeks. *N* = 3. (C) qPCR was used to detect the relative mRNA levels (normalized to GAPDH) of PPARγ from the tendon lesions at 3 weeks. *N* = 5; **p < 0.01, ****p < 0.0001. (D) WB analysis was used to detect the expression of PPARγ in the tendon lesions at 3 weeks. *N* = 3; *p < 0.05, ****p < 0.0001. (E) IF staining was used to detect the positive cells of PPARγ (red), co‐localized with PDGFR‐α (green), in the tendon lesions at 3 weeks. *N* = 5; scale bar = 250 μm (original magnification) and 50 μm (insert magnification of the boxed area, 5.0×); *p < 0.05, ***p < 0.001, ****p < 0.0001. (F) Schematic depiction of the intervention protocols for animal experiments. The mice were randomly assigned into six groups: (i) DMSO+ov‐NC (burn/tenotomy with intraperitoneally vehicle [DMSO] injection, along with locally tendon overexpression vehicle injection), (ii) PPARγ+ (burn/tenotomy with intraperitoneally Rosiglitazone injection, a promotor for PPARγ, along with locally tendon overexpression vehicle injection), (iii) PPARγ+/KLF2+ (burn/tenotomy with intraperitoneally Rosiglitazone injection, along with locally tendon ov‐KLF2 injection), (iv) DMSO+sh‐NC (burn/tenotomy with intraperitoneally vehicle injection, along with locally tendon downregulation vehicle injection), (v) PPARγ− (burn/tenotomy with intraperitoneally GW9662 injection, an inhibitor for PPARγ, along with locally tendon downregulation vehicle injection) and (vi) PPARγ−/KLF2− (burn/tenotomy with intraperitoneally GW9662 injection, along with locally tendon sh‐KLF2 injection). (G) Micro‐CT was used to detect HO formation at 10 weeks in the tendon lesions. The volume of HO was quantified from each group. *N* = 5; **p < 0.01, ****p < 0.0001. (H) IHC staining was used to detect osteogenesis by the positive cells of RUNX2 in the tendon lesions at 3 weeks. *N* = 5; scale bar = 250 μm (original magnification) and 50 μm (insert magnification of the boxed area, 5.0×); **p < 0.01, ****p < 0.0001. IF, immunofluorescence; micro‐CT, micro‐computed tomography; qPCR, quantitative real‐time reverse transcriptase polymerase chain reaction; WB, western blot.

To investigate the role of PPARγ in trauma‐induced HO, PPARγ expression was evaluated first. Between the two groups (i) SHAM and (ii) BTT, both the heat map (Figure 3A) and the volcano map (Figure 3B) of the high‐throughput whole transcriptome sequencing of tendon lesions at 3 weeks showed a significantly reduced PPARγ expression in BTT group, as well as qRT‐PCR (PPARγ; Figure 3C), WB analysis (PPARγ; Figure 3D) and IF staining (PPARγ, co‐localized with PDGFRα; Figure 3E). Further, the effects of PPARγ on mature HO and chondro‐osteogenesis indicators between burn/tenotomy mice and the same model administrated with PPARγ activator (Rosiglitazone) or inhibitor (GW9662) were compared (Figure 3F). Between the two groups (i) DMSO+ov‐NC, and (ii) PPARγ+, micro‐CT (Figure 3G), H&E staining (Figure S1A), SOFG staining (Figure S1B) and IHC staining (RUNX2; Figure 3H) all confirmed that PPARγ+ group had markedly decreased matured HO at 10 weeks and chondro‐osteogenesis at 3 weeks post‐tendon injury. However, between the two groups (i) DMSO+sh‐NC, and (ii) PPARγ−, opposite effects were observed (Figure 3G,H, Figure S1A,B). Taken together, these results suggested that PPARγ participated in trauma‐induced HO formation in burn/tenotomy mice.

Next, to explore whether the effects of PPARγ on trauma‐induced HO were regulated by KLF2, the PPARγ expression was tested between the two groups, sh‐NC and KLF2−, as well as ov‐NC and KLF2+. qRT‐PCR, WB analysis and IF staining (Figure 3C–E) all showed increased PPARγ expression in KLF2− group when compared with sh‐NC group, whereas reduced PPARγ expression in KLF2+ group when compared with ov‐NC group. Furthermore, mature HO and chondro‐osteogenesis indicators were compared between burn/tenotomy mice administrated with PPARγ activator (Rosiglitazone) or inhibitor (GW9662), and the same model co‐with AAVs overexpressed or down‐regulated KLF2 (Figure 3F). As expected, when compared with PPARγ+ group, KLF2 overexpression in PPARγ+/KLF2+ group reverse the effects of PPARγ promotion on mature HO and chondro‐osteogenesis indicators (Figure 3G,H, Figure S1A,B); and similar reversed results were shown for KLF2 suppression between PPARγ‐ and PPARγ−/KLF2− groups (Figure 3G,H, Figure S1A,B). Taken together, the results suggested that the KLF2/PPARγ axis plays a key role in trauma‐induced HO formation.

### 3.4 KLF2/PPARγ axis participated in trauma‐induced HO formation by regulating mitochondrial dysfunction in burn/tenotomy mice

Based on previous reports indicating that PPARγ affects mitochondrial function,^19^ it was investigated whether KLF2/PPARγ mediated mitochondrial dysfunction participates in trauma‐induced HO formation.

**FIGURE 4:**
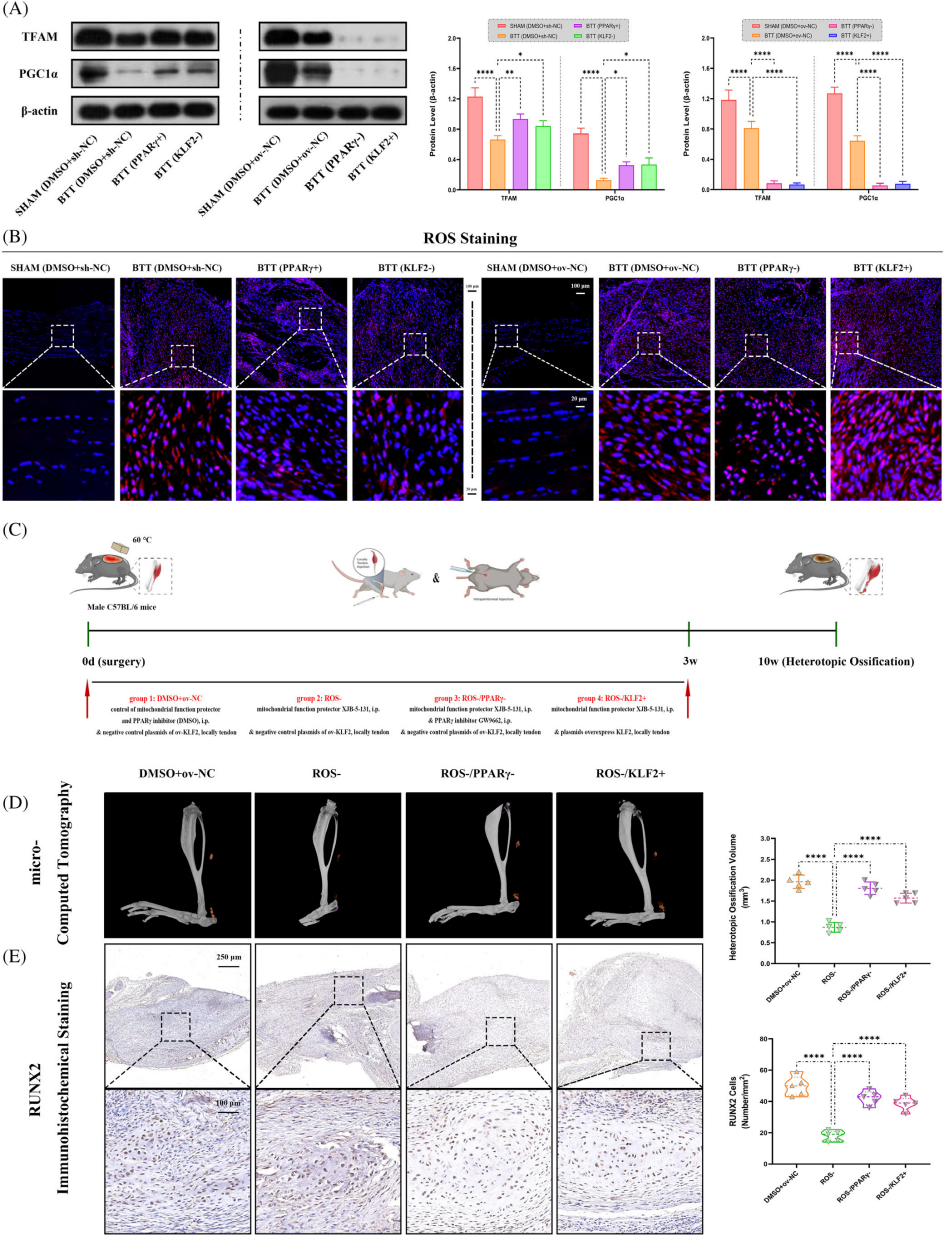
Role of mitochondrial dysfunction under KLF2/PPARγ pathway in trauma‐induced HO formation in burn/tenotomy mice. (A) WB analysis was used to detect the expression of mitochondrial function‐related proteins in the tendon lesions at 3 weeks. *N* = 3; *p < 0.05, **p < 0.01, ****p < 0.0001. (B) ROS staining was used to detect mitochondrial dysfunction in the tendon lesions at 3 weeks. *N* = 5; scale bar = 100 μm (original magnification) and 20 μm (insert magnification of the boxed area, 5.0×). (C) Schematic depiction of the intervention protocols for animal experiments. The mice were randomly assigned into four groups: (i) DMSO+ov‐NC (burn/tenotomy with intraperitoneally vehicle injection, along with locally tendon overexpression vehicle injection), (ii) ROS− (burn/tenotomy with intraperitoneally XJB‐5‐131 injection, a mitochondrial function protector, ROS and electron scavenger, along with locally tendon overexpression vehicle injection), (iii) ROS−/PPARγ− (burn/tenotomy with intraperitoneally XJB‐5‐131 and GW9662 injection, along with locally tendon overexpression vehicle injection) and (iv) ROS−/KLF2+ (burn/tenotomy with intraperitoneally XJB‐5‐131 injection, along with locally tendon ov‐KLF2 injection). (D) Micro‐CT was used to detect HO formation at 10 weeks in the tendon lesions. The volume of HO was quantified from each group. *N* = 5; ****p < 0.0001. (E) IHC staining was used to detect osteogenesis by the positive cells of RUNX2 in the tendon lesions at 3 weeks. *N* = 5; scale bar = 250 μm (original magnification) and 50 μm (insert magnification of the boxed area, 5.0×); ****p < 0.0001. HO, heterotopic ossification; IHC, immunohistochemistry; micro‐CT, micro‐computed tomography; ROS, reactive oxygen species; WB, western blot.

To investigate the role of mitochondrial in trauma‐induced HO, mitochondrial function was evaluated first. Between the two groups (i) SHAM and (ii) BTT, WB analysis (TFAM and PGC1α; Figure 4A), and reactive oxygen species (ROS) staining (Figure 4B) showed more mitochondrial dysfunction in BTT group. Further, the effects of mitochondrial function on trauma‐induced HO between burn/tenotomy mice and the same model administrated with mitochondrial function protector (XJB‐5‐131, ROS and electron scavenger) were compared (Figure 4C). Between the two groups, (i) DMSO+ov‐NC, and (ii) ROS‐, micro‐CT (Figure 4D), H&E staining (Figure S2A), SOFG staining (Figure S2B) and IHC staining (RUNX2; Figure 4E) all confirmed that ROS− group had markedly decreased matured HO at 10 weeks and chondro‐osteogenesis at 3 weeks post‐tendon injury. These results indicate that mitochondrial dysfunction participates in the trauma‐induced HO formation process in burn/tenotomy mice.

Next, to explore whether regulation of mitochondrial dysfunction in trauma‐induced HO was mediated by the KLF2/PPARγ axis, the mitochondrial function was tested among the three groups, DMSO+sh‐NC and PPARγ+ and KLF2−, as well as DMSO+ov‐NC and PPARγ− and KLF2+. WB analysis and ROS staining (Figure 4A,B) showed reduced mitochondrial dysfunction in both PPARγ+ and KLF2− groups when compared with DMSO+sh‐NC group, whereas increased mitochondrial dysfunction in both PPARγ− and KLF2+ groups when compared with DMSO+ov‐NC group. Furthermore, mature HO and chondro‐osteogenesis indicators were compared between burn/tenotomy mice administrated with mitochondrial function protector (XJB‐5‐131, ROS and electron scavenger), and the same model co‐with PPARγ inhibitor (GW9662) or AAVs overexpressed KLF2 (Figure 4C). As expected, when compared with ROS‐ group, both PPARγ inhibition in ROS−/PPARγ− group and KLF2 overexpression in ROS−/KLF2+ group reverse the effects of mitochondrial function protector on trauma‐induced HO (Figure 4D,E, Figure S2A,B). Taken together, the KLF2/PPARγ axis‐dependent mitochondrial dysfunction and played a key role in trauma‐induced HO formation.

### 3.5 The expression of KLF2 was elevated in TSPCs during osteogenic induction

**FIGURE 5:**
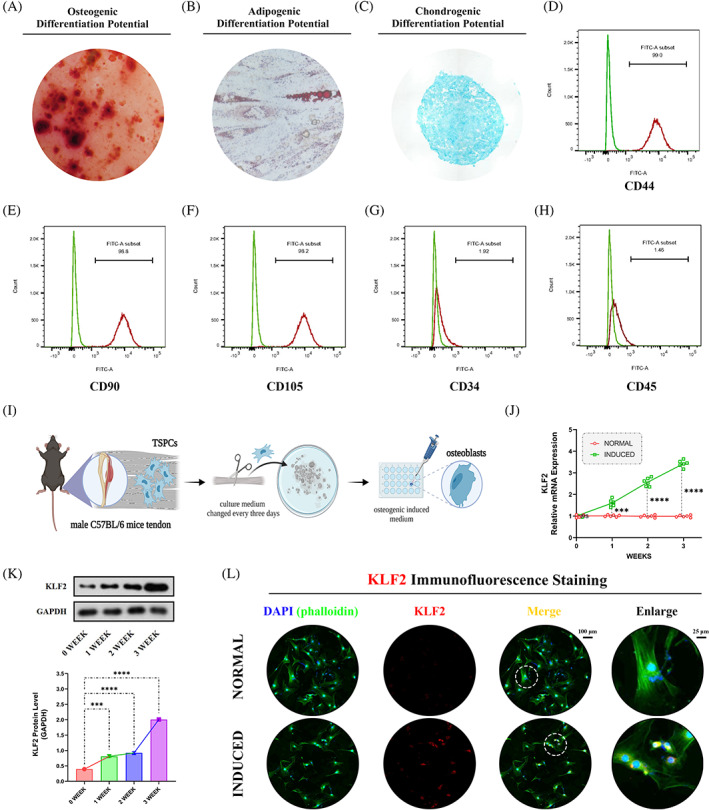
Increased expression of KLF2 in vitro after osteogenic induction for TSPCs. (A) ARS staining was used to detect TSPCs osteogenic induction at 3 weeks. (B) Oil Red O staining was used to detect TSPCs adipogenic differentiation at 3 weeks. (C) Alcian blue staining was used to detect TSPCs chondrogenic differentiation at 3 weeks. (D–H) Flow cytometry was used to characterize the MSC properties of TSPCs by identifying cell surface markers. (I) Schematic depiction of the establishment of TSPCs osteogenic induction. The cells were randomly divided into two groups: (i) NORMAL (sham medium) and (ii) INDUCED (osteo‐inductive medium). (J) qPCR was used to detect the relative mRNA levels (normalized to GAPDH) of KLF2 in the osteogenic‐induced TSPCs. *N* = 6; ***p < 0.001, ****p < 0.0001, ns = no significance. (K) WB analysis was used to detect the expression of KLF2 in the osteogenic‐induced TSPCs. *N* = 3; ***p < 0.001, ****p < 0.0001. (L) IF staining was used to detect the expression of KLF2 (red), co‐stained with phalloidin (green) and DAPI (blue) in the osteogenic‐induced TSPCs. *N* = 6, scale bar = 100 μm (original magnification) and 25 μm (insert magnification of the boxed area, 4.0×). ARS, Alizarin red S; IF, immunofluorescence; MSC, mesenchymal stem cell; qPCR, quantitative real‐time reverse transcriptase polymerase chain reaction; TSPC, tendon stem/progenitor cell; WB, western blot.

Subsequently, these findings were validated in vitro using TSPCs to further explore the role of KLF2 in the regulation of stem cell osteogenesis. TSPCs were successfully obtained, and showed osteogenic, adipogenic and chondrogenic differentiation potential after 3 weeks of induction (Figure 5A–C). The MSC properties of TSPCs were characterized by identifying cell surface markers. TSPCs were positive for the mesenchymal markers cluster of differentiation 44 (CD44, 99.0%), CD90 (98.8%) and CD105 (98.2%), but negative for the endothelial progenitor marker CD34 (1.92%) and haematopoietic marker CD45 (1.46%) in flow cytometric analysis (Figure 5D–H). These results suggest that TSPCs were successfully isolated and had good osteogenic differentiation ability. Osteogenic induction medium (Cyagen) was used to culture TSPCs (in vitro cell model; Figure 5I). Between the two groups (i) NORMAL and (ii) INDUCED, qPCR and WB analysis (Figure 5J,K) showed gradually elevated levels of KLF2 in osteogenic‐induced TSPCs at weeks 1, 2 and 3, respectively, which was also confirmed by IF staining (KLF2; Figure 5L) at 3 weeks.

### 3.6 KLF2 promoted osteogenic differentiation of TSPCs

**FIGURE 6:**
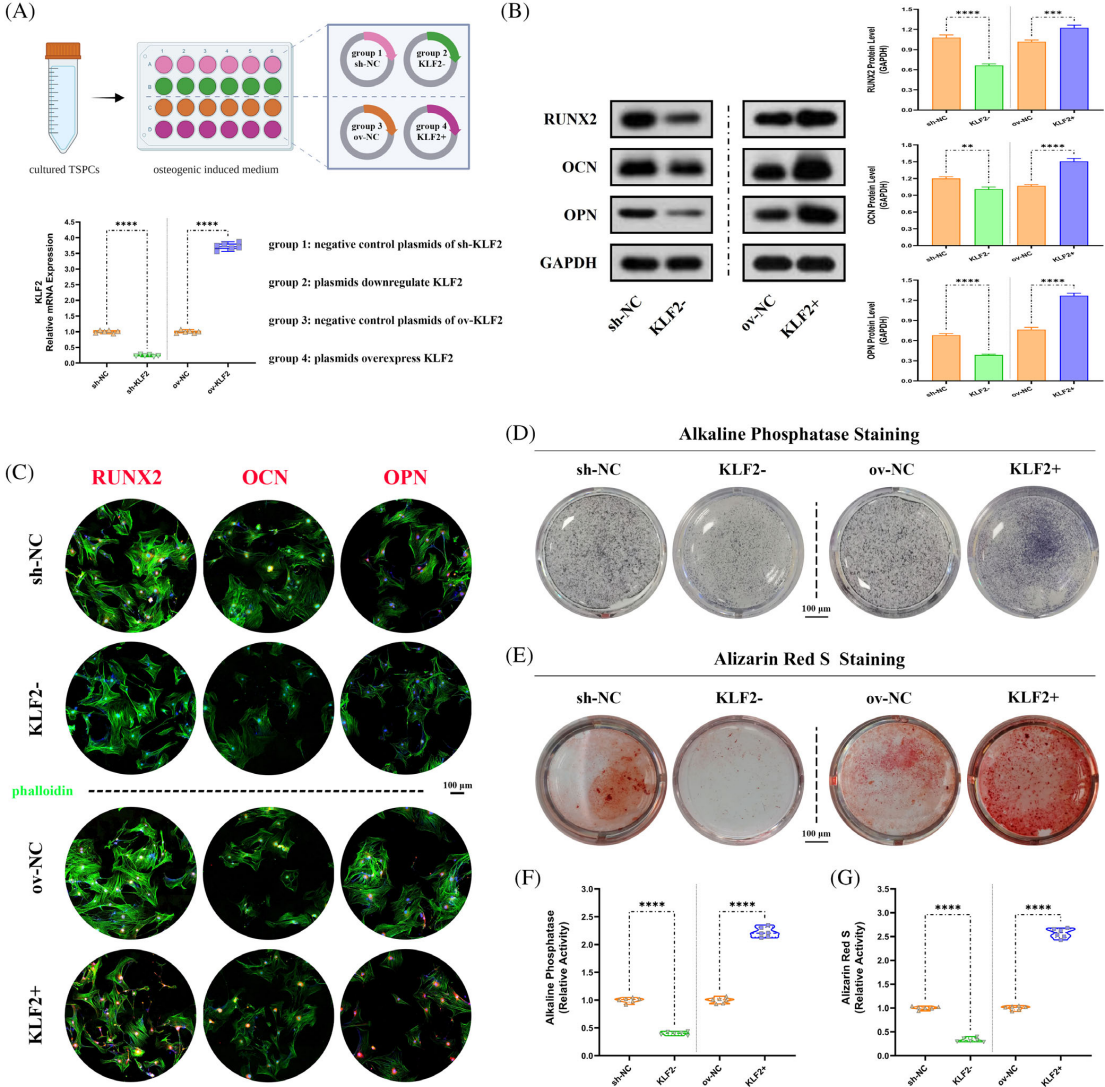
Reduced osteogenesis after KLF2 inhibition in vitro for osteogenic induced TSPCs. (A) Schematic depiction of the intervention protocols for TSPCs experiments. The cells were randomly assigned into four groups: (i) sh‐NC (osteo‐inductive medium with downregulation vehicle [plasmids harbouring no downregulation sequence]), (ii) KLF2− (osteo‐inductive medium with sh‐KLF2 [plasmids downregulate KLF2]), (iii) ov‐NC (osteo‐inductive medium with overexpression vehicle [plasmids harbouring no overexpression sequence]) and (iv) KLF2+ (osteo‐inductive medium with ov‐KLF2 [plasmids overexpress KLF2]). qPCR was used to detect the relative mRNA levels (normalized to GAPDH) of KLF2 in the osteogenic‐induced TSPCs after using plasmids. *N* = 6; ****p < 0.0001. (B) WB analysis was used to detect the expression of osteogenesis‐related proteins in the osteogenic‐induced TSPCs. *N* = 3; **p < 0.01, ***p < 0.001, ****p < 0.0001. (C) IF staining was used to detect the expression of RUNX2 (red), OCN (red) and OPN (red), co‐stained with phalloidin (green) and DAPI (blue), in the osteogenic‐induced TSPCs. *N* = 6, scale bar = 100 μm. (D, F) ALP and (E, G) ARS staining were used to detect the osteogenesis of TSPCs. *N* = 6; ****p < 0.0001. ALP, alkaline phosphatase; ARS, Alizarin red S; IF, immunofluorescence; qPCR, quantitative real‐time reverse transcriptase polymerase chain reaction; TSPC, tendon stem/progenitor cell; WB, western blot.

After confirmation that elevated KLF2 levels were detectable during osteogenic differentiation of TSPCs, the effects of KLF2 on osteogenesis in vitro were examined between osteogenic‐induced TSPCs and the same model administrated with plasmids down‐regulated or overexpressed KLF2 (Figure 6A). Between the two groups (i) sh‐NC and (ii) KLF2−, WB analysis (Runx2, OCN and OPN; Figure 6B), cell IF staining (Runx2, OCN and OPN; Figure 6C) and ALP and ARS staining (Figure 6D–G) confirmed that KLF2− group had markedly decreased osteogenesis. However, between the two groups (i) ov‐NC and (ii) KLF2+, opposite effects were observed (Figure 6B–G). In addition, as for adipogenic differentiation of TSPCs, WB analysis (sterol regulatory element‐binding protein 1 [SREBP1], Figure S3A), cell IF staining (SREBP1, Figure S3B) and Oil Red O staining (Figure S3C) all showed markedly increased adipogenesis for KLF2− group when compared with sh‐NC group, and decreased adipogenesis for KLF2+ group when compared with ov‐NC group. These results indicate that inhibition of KLF2 could attenuate osteogenesis and promote adipogenesis in TSPCs.

### 3.7 PPARγ participated in the regulation of KLF2 in osteogenic differentiation of TSPCs

From in vivo study above, KLF2/PPARγ axis has been approved to play a key role in the process of trauma‐induced HO formation. Here, the potential relationship between KLF2 and PPARγ on TSPCs osteogenesis was further investigated in vitro.

**FIGURE 7:**
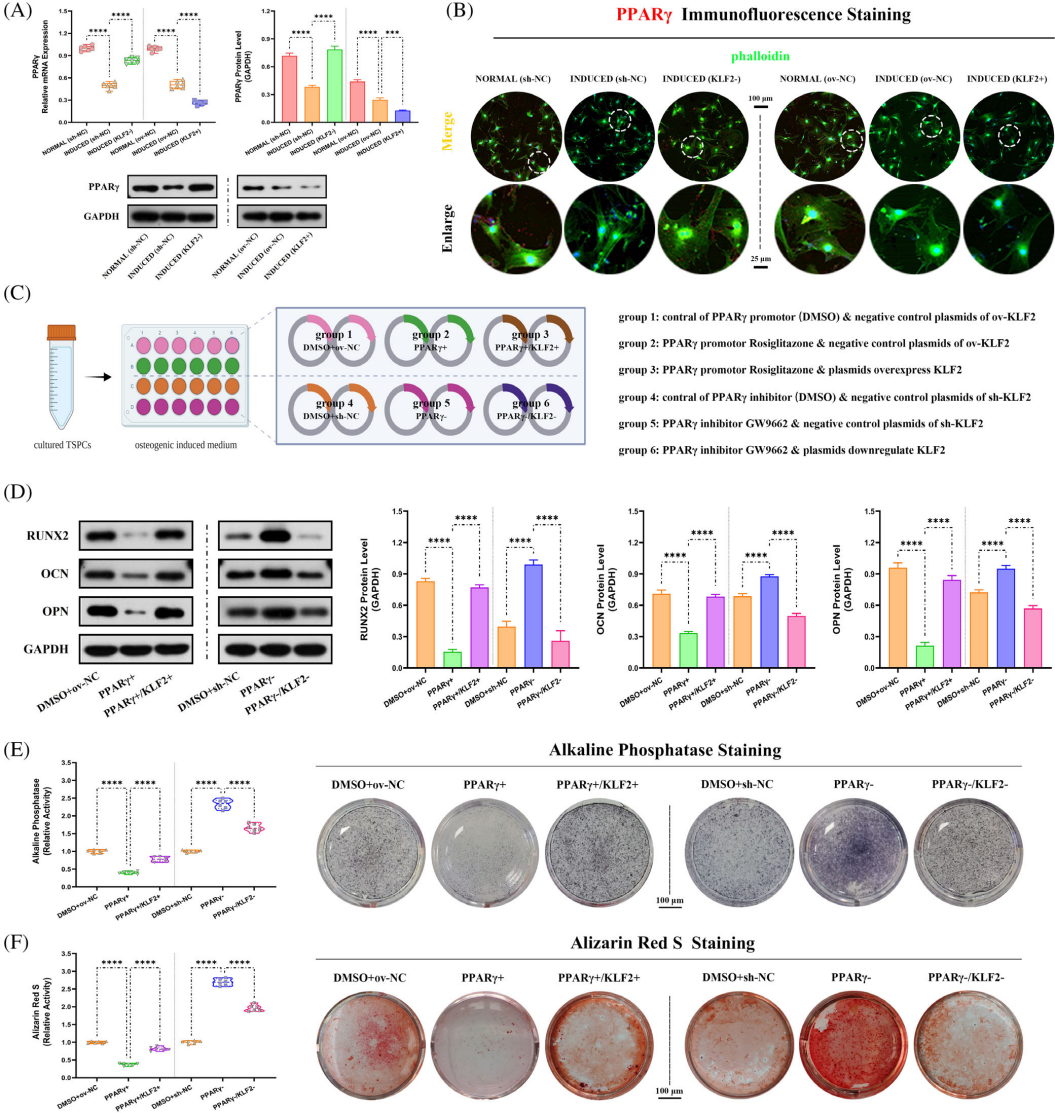
Role of PPARγ in regulation of KLF2 on osteogenesis in osteogenic induced TSPCs. (A) qPCR and WB analysis were used to detect the relative mRNA (normalized to GAPDH) and protein expression of PPARγ in the osteogenic‐induced TSPCs. *N* = 6; ***p < 0.001, ****p < 0.0001. (B) IF staining was used to detect the expression of PPARγ (red), co‐stained with phalloidin (green) and DAPI (blue), in the osteogenic‐induced TSPCs. *N* = 6, scale bar = 100 μm (original magnification) and 25 μm (insert magnification of the boxed area, 4.0×). (C) Schematic depiction of the establishment of TSPCs osteogenic induction. The cells were randomly assigned into six groups: (i) DMSO+ov‐NC (osteo‐inductive medium with drug vehicle [DMSO], along with overexpression vehicle), (ii) PPARγ+ (osteo‐inductive medium with Rosiglitazone, along with overexpression vehicle), (iii) PPARγ+/KLF2+ (osteo‐inductive medium with Rosiglitazone, along with ov‐KLF2), (iv) DMSO+sh‐NC (osteo‐inductive medium with drug vehicle, along with downregulation vehicle), (v) PPARγ− (osteo‐inductive medium with GW9662, along with downregulation vehicle) and (vi) PPARγ−/KLF2− (osteo‐inductive medium with GW9662, along with sh‐KLF2). (D) WB analysis was used to detect the expression of osteogenesis‐related proteins in the osteogenic‐induced TSPCs. *N* = 3; ****p < 0.0001. (E, F) ALP and ARS staining were used to detect the osteogenesis of TSPCs. *N* = 6; ****p < 0.0001. ALP, alkaline phosphatase; ARS, Alizarin red S; IF, immunofluorescence; qPCR, quantitative real‐time reverse transcriptase polymerase chain reaction; TSPC, tendon stem/progenitor cell; WB, western blot.

To investigate the role of PPARγ on osteogenic differentiation of TSPCs, PPARγ expression was evaluated first. Between the two groups (i) NORMAL and (ii) INDUCED, qRT‐PCR and WB analysis (PPARγ; Figure 7A) and IF staining (PPARγ; Figure 7B) showed a significantly reduced PPARγ expression in INDUCED group. Further, the effects of PPARγ on osteogenesis in vitro between osteogenic induced TSPCs and the same model administrated with PPARγ activator (Rosiglitazone) or inhibitor (GW9662) were compared (Figure 7C). Between the two groups, (i) DMSO+ov‐NC, and (ii) PPARγ+, WB analysis (Runx2, OCN and OPN; Figure 7D), cell IF staining (Runx2, OCN and OPN; Figure S4A–C) and ALP and ARS staining (Figure 7E,F) all revealed that PPARγ+ group had markedly decreased osteogenic differentiation. However, between the two groups (i) DMSO+sh‐NC, and (ii) PPARγ−, opposite effects were observed (Figure 7D–F, Figure S4A–C). In addition, as for adipogenic differentiation of TSPCs, WB analysis (SREBP1, Figure S5A), cell IF staining (SREBP1; Figure S5B) and Oil Red O staining (Figure S5C) all showed markedly increased adipogenesis for PPARγ+ group when compared with DMSO+ov‐NC group, and decreased adipogenesis for PPARγ− group when compared with DMSO+sh‐NC group. These results suggested that activation of PPARγ could attenuate osteogenesis and promote adipogenesis in TSPCs.

Subsequently, to explore whether the effects of PPARγ on osteogenic differentiation of TSPCs were regulated by KLF2, the PPARγ expression was tested between the two groups, sh‐NC and KLF2−, as well as ov‐NC and KLF2+. qRT‐PCR, WB analysis and IF staining (Figure 7A,B) all showed increased PPARγ expression in KLF2− group when compared with sh‐NC group, whereas reduced PPARγ expression in KLF2+ group when compared with ov‐NC group. Furthermore, osteogenesis in vitro was compared between osteogenic induced TSPCs administrated with PPARγ activator (Rosiglitazone) or inhibitor (GW9662), and the same model co‐with plasmids overexpressed or down‐regulated KLF2 (Figure 7C). As expected, when compared with PPARγ+ group, KLF2 overexpression in PPARγ+/KLF2+ group reverse the effects of PPARγ promotion on osteogenesis (Figure 7D–F, Figure S4A‐C); and similar reversed results were shown for KLF2 suppression between PPARγ− and PPARγ−/KLF2− groups (Figure 7D–F, Figure S4A–C). In addition, as for adipogenic differentiation of TSPCs, WB analysis (SREBP1; Figure S5A), cell IF staining (SREBP1, Figure S5B) and Oil Red O staining (Figure S5C) all showed markedly reversed adipogenesis for PPARγ+ and PPARγ+/KLF2+ groups, as well as PPARγ− and PPARγ−/KLF2− groups. Taken together, the KLF2/PPARγ axis played a key role in the osteogenesis and adipogenesis of TSPCs.

### 3.8 KLF2/PPARγ axis participated in osteogenic differentiation by regulating mitochondrial dysfunction in TSPCs

From the in vivo study above, the KLF2/PPARγ axis‐mediated mitochondrial dysfunction and played a key role in trauma‐induced HO formation. Here, we further investigate whether this pathway participated in osteogenesis of TSPCs in vitro.

**FIGURE 8:**
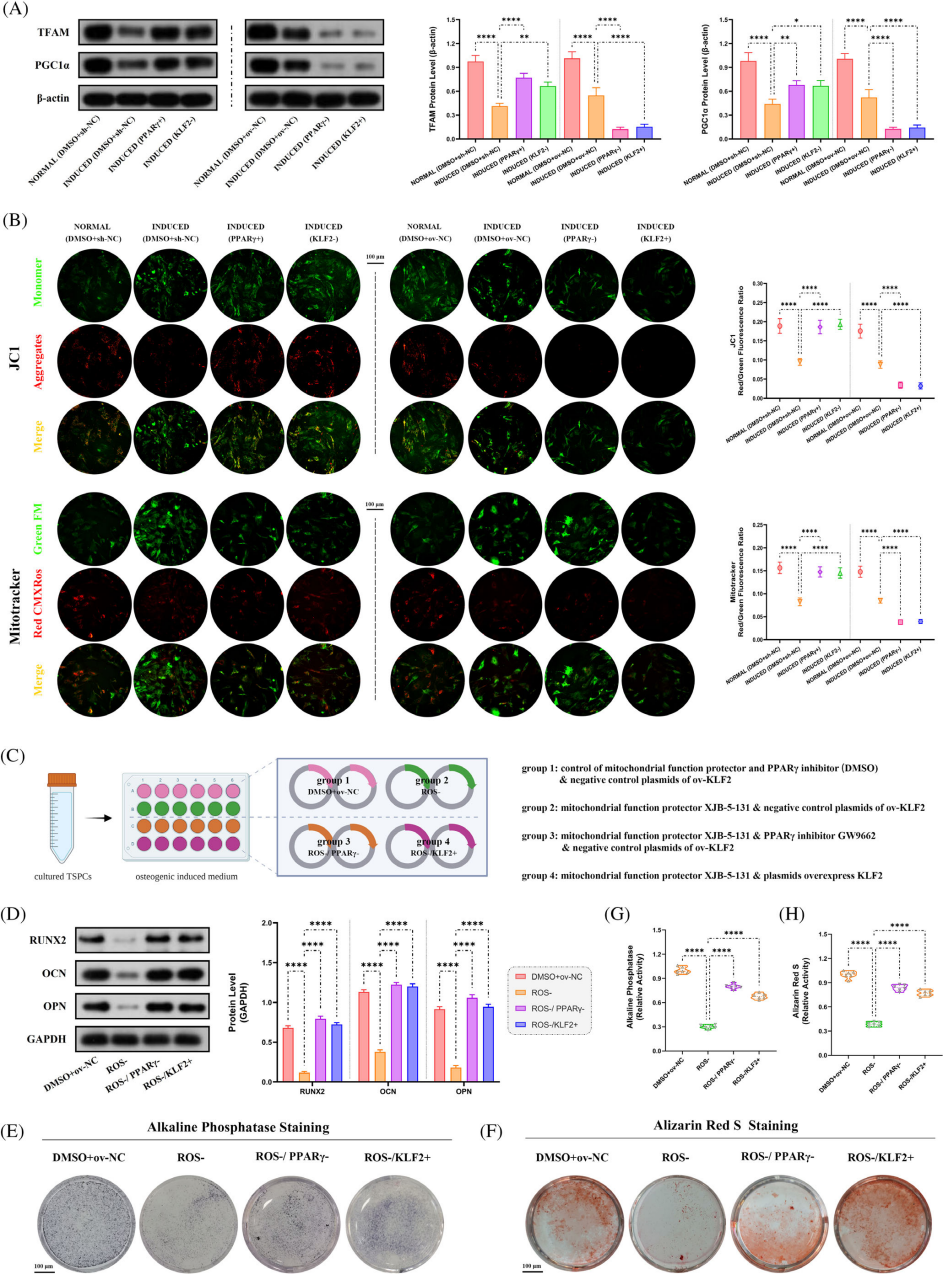
Role of mitochondrial dysfunction under KLF2/PPARγ pathway on osteogenesis in osteogenic‐induced TSPCs. (A) WB analysis was used to detect the expression of mitochondrial function‐related proteins in the osteogenic‐induced TSPCs. *N* = 3; *p < 0.05, **p < 0.01, ****p < 0.0001. (B) JC1 and mitotracker staining were used to detect mitochondrial dysfunction in the osteogenic‐induced TSPCs. *N* = 6; ****p < 0.0001; scale bar = 100 μm. (C) Schematic depiction of the establishment of TSPCs osteogenic induction. The cells were randomly assigned into four groups: (i) DMSO+ov‐NC (osteo‐inductive medium with drug vehicle, along with overexpression vehicle), (ii) ROS− (osteo‐inductive medium with XJB‐5‐131, along with overexpression vehicle), (iii) ROS−/PPARγ− (osteo‐inductive medium with XJB‐5‐131 and GW9662, along with overexpression vehicle) and (iv) ROS−/KLF2+ (osteo‐inductive medium with XJB‐5‐131, along with ov‐KLF2). (D) WB analysis was used to detect the expression of osteogenesis‐related proteins in the osteogenic‐induced TSPCs. *N* = 3; ****p < 0.0001. (E, G) ALP and (F, H) ARS staining were used to detect the osteogenesis of TSPCs. *N* = 6; ****p < 0.0001. ALP, alkaline phosphatase; ARS, Alizarin red S; ROS, reactive oxygen species; TSPC, tendon stem/progenitor cell; WB, western blot.

To investigate the role of mitochondrial on osteogenic differentiation of TSPCs, mitochondrial function was evaluated first. Between the two groups (i) NORMAL and (ii) INDUCED, WB analysis (TFAM and PGC1α; Figure 8A), JC1 and Mitotracker staining (Figure 8B), cell IF staining (TOMM20, a marker to observe the morphological changes of mitochondria; Figure S6A), and the content of ATP level (Figure S6B) showed more mitochondrial dysfunction in INDUCED group. Further, the effects of mitochondrial function on osteogenesis in vitro between osteogenic induced TSPCs and the same model administrated with mitochondrial function protector (XJB‐5‐131, ROS and electron scavenger) were compared (Figure 8C). Between the two groups, (i) DMSO+ov‐NC, and (ii) ROS−, WB analysis (Runx2, OCN and OPN; Figure 8D), cell IF staining (Runx2, OCN and OPN; Figure S7A–C) and ALP and ARS staining (Figure 8E–H) all revealed that ROS− group had markedly decreased osteogenic differentiation. These results indicated that mitochondrial dysfunction participates in the osteogenic differentiation process in TSPCs.

Next, to explore whether the regulation of mitochondrial function in TSPCs osteogenesis was mediated by the KLF2/PPARγ axis, the mitochondrial function was tested among the three groups, DMSO+sh‐NC and PPARγ+ and KLF2−, as well as DMSO+ov‐NC and PPARγ− and KLF2+. WB analysis, JC1 and Mitotracker staining, cell IF staining (TOMM20) and the content of ATP level (Figure 8A,B and Figure S6A,B) all showed reduced mitochondrial dysfunction in both PPARγ+ and KLF2− groups when compared with DMSO+sh‐NC group, whereas increased mitochondrial dysfunction in both PPARγ− and KLF2+ groups when compared with DMSO+ov‐NC group. Furthermore, osteogenesis in vitro was compared between osteogenic induced TSPCs administrated with mitochondrial function protector (XJB‐5‐131, ROS and electron scavenger), and the same model co‐with PPARγ inhibitor (GW9662) or plasmids overexpressed KLF2 (Figure 8C). As expected, when compared with ROS− group, both PPARγ inhibition in ROS−/PPARγ− group and KLF2 overexpression in ROS−/KLF2+ group reverse the effects of mitochondrial function protector on osteogenesis (Figure 8D–H, Figure S7A–C). Taken together, mitochondrial dysfunction dependent by the KLF2/PPARγ axis plays a key role in the osteogenesis process of TSPCs.

### 3.9 KLF2/PPARγ axis regulated mitochondrial function and ROS production by affecting mitochondrial redox balance

With the above experimental results, we demonstrate that reducing the level of ROS leads to a reduction in trauma‐induced HO and osteogenic differentiation of TSPCs, as well as a negative regulatory relationship between PPARγ, ROS and mitochondrial membrane potential. Inhibition of mitochondrial anti‐oxidative damages components of electron transport chain, decreases mitochondrial membrane potential and promotes production of ROS.^22^, ^23^, ^24^ Emerging evidence has also shown that PPARγ plays an oncogenic role via mitochondrial antioxidative function, such as downregulating PPARγ causes downregulation of key ROS scavenger proteins, superoxide dismutase 2 (SOD2) and catalase (CAT).^25^, ^26^ SOD2, the primary mitochondrial oxidative scavenger, plays a crucial role during the regulation of mitochondrial ROS by catalysing O2− conversation to H_2_O_2_, and can be further neutralized by conversion to H_2_O through the CAT, both of which (SOD2 and CAT) are the vital proteins to maintain the mitochondrial redox balance.^24^, ^27^, ^28^

**FIGURE 9:**
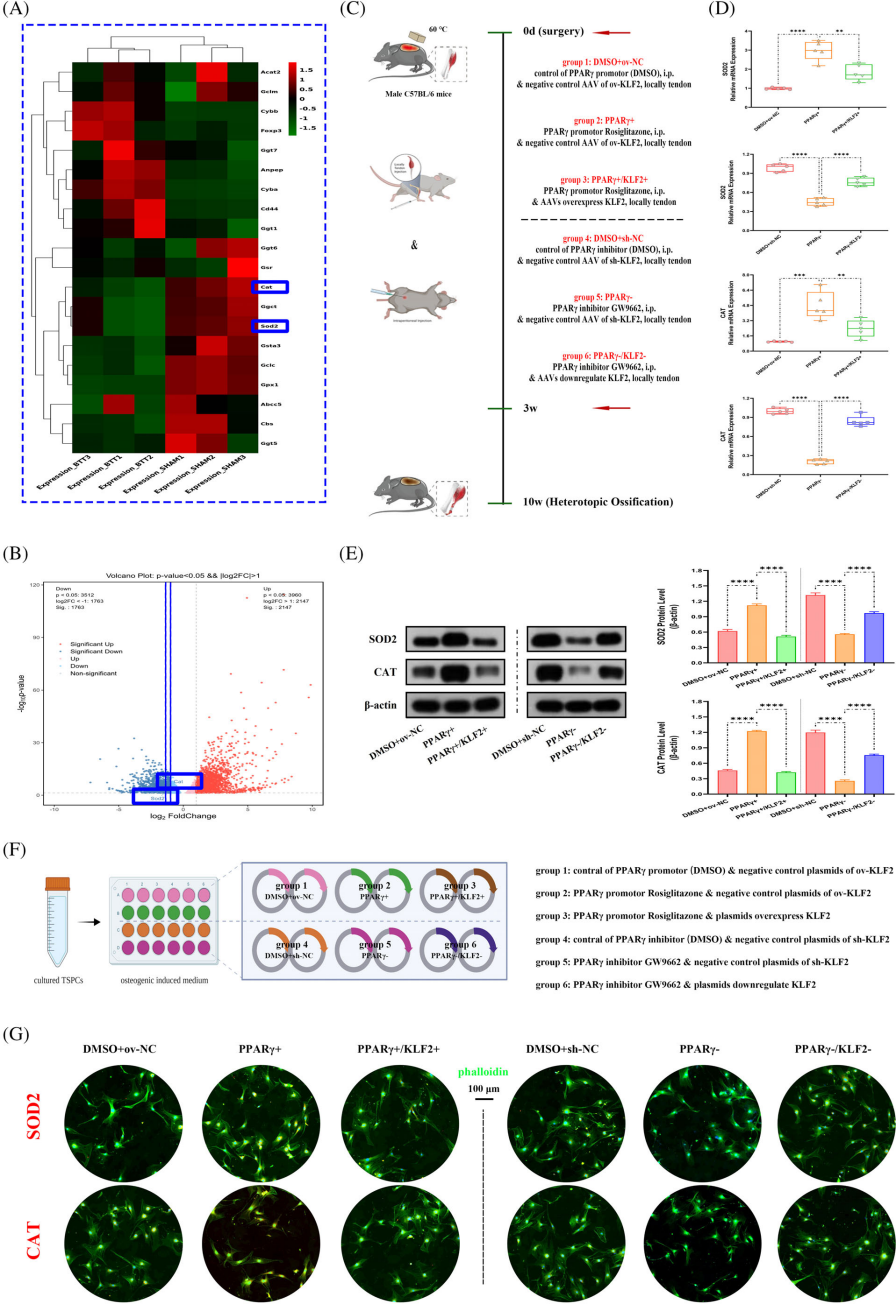
Mitochondrial redox balance regulated by KLF2/PPARγ axis in both burn/tenotomy mice and osteogenic induced TSPCs. (A, B) High‐throughput whole‐transcriptome sequencing was performed and showed by heat map and volcano plot in the tendon lesions at 3 weeks. *N* = 3. (C) Schematic depiction of the intervention protocols for animal experiments. (D) qPCR was used to detect the relative mRNA levels (normalized to GAPDH) of SOD2 and CAT from the tendon lesions at 3 weeks. *N* = 5; **p < 0.01, ***p < 0.001, ****p < 0.0001. (E) WB analysis was used to detect the expression of SOD2 and CAT in the tendon lesions at 3 weeks. *N* = 3; ****p < 0.0001. (F) Schematic depiction of the establishment of TSPCs osteogenic induction. (G) IF staining was used to detect the expression of SOD2 (red) and CAT (red), co‐stained with phalloidin (green) and DAPI (blue), in the osteogenic‐induced TSPCs. *N* = 6, scale bar = 100 μm (original magnification). IF, immunofluorescence; qPCR, quantitative real‐time reverse transcriptase polymerase chain reaction; TSPC, tendon stem/progenitor cell; WB, western blot.

Further, to investigate how KLF2/PPARγ axis regulated mitochondrial function and ROS production in trauma‐induced HO formation, SOD2 and CAT expressions were evaluated first. In vivo, between the two groups (i) SHAM and (ii) BTT, both the heat map (Figure 9A) and the volcano map (Figure 9B) of the high‐throughput whole transcriptome sequencing of tendon lesions at 3 weeks showed a significantly reduced SOD2 and CAT expression in BTT group. Next, to explore whether SOD2 and CAT were regulated by KLF2/PPARγ axis, the SOD2 and CAT expression was tested among the three groups (Figure 9C *)*, DMSO+ov‐NC, PPARγ+ and PPARγ+/KLF2+, as well as DMSO+sh‐NC, PPARγ− and PPARγ−/KLF2−. qPCR (SOD2 and CAT; Figure 9D) and WB analysis (SOD2 and CAT; Figure 9E) showed increased SOD2 and CAT expression in PPARγ+ group when compared with DMSO+ov‐NC group, and reversed in PPARγ+/KLF2+ group, whereas reduced SOD2 and CAT expression in PPARγ− group when compared with DMSO+sh‐NC group, and reversed in PPARγ−/KLF2− group. In addition, in vitro (Figure 9F *)*, qPCR and WB analysis (SOD2 and CAT; Figure S8A,B) and cell IF staining (SOD2 and CAT; Figure 9G) all showed similar results. Therefore, we suggested that KLF2/PPARγ axis regulated mitochondrial function and ROS production by influencing the expression levels of SOD2 and CAT (redox balance) in mitochondria, and ultimately affecting the formation of trauma‐induced HO.

## 4 DISCUSSION

**FIGURE 10:**
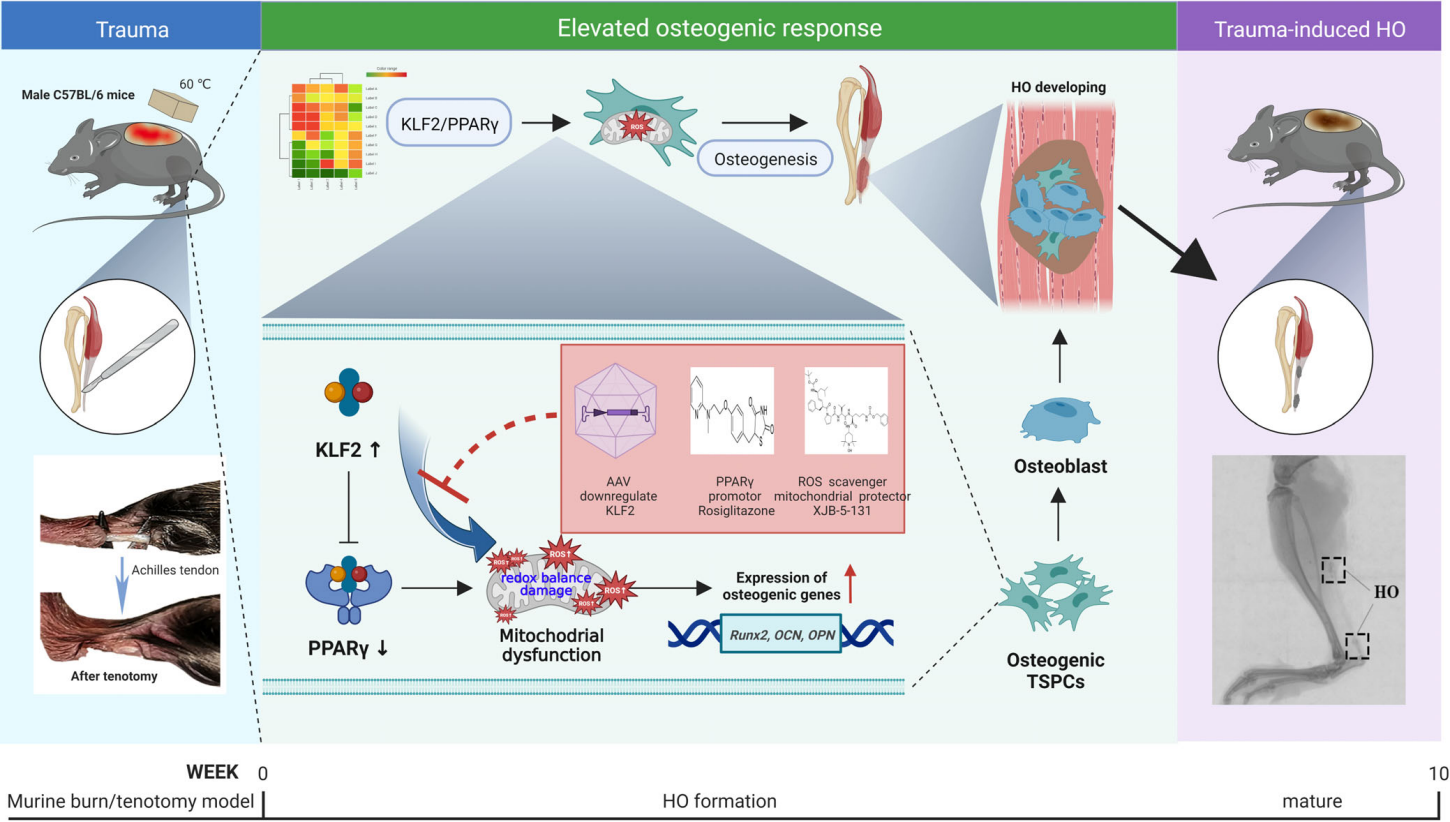
Graphical summary of effects and mechanisms of KLF2/PPARγ pathway‐mediated mitochondrial dysfunction that regulate traumatic HO progression. After burn/tenotomy, tendon stem/progenitor cells (TSPCs) accumulate in the tendon lesions, and the elevated expression of KLF2 further promotes PPARγ‐dependent mitochondrial dysfunction and ROS production by affecting redox balance in the TSPCs, resulting in enhanced expression of osteogenic genes and inducing osteogenesis differentiation, and finally leads to HO formation. Further, all of the AAVs downregulate KLF2, PPARγ promotor Rosiglitazone and ROS scavenger XJB‐5‐131 could suppress mitochondrial dysfunction involved in these biological processes, thereby inhibiting the HO formation. AAV, adeno‐associated virus; HO, heterotopic ossification; ROS, reactive oxygen species.

The complex pathological nature of trauma‐induced HO has received considerable attention, particularly with respect to the crucial role of TSPCs in osteogenic differentiation. To our knowledge, this is the first study to reveal the osteogenic commitment of TSPCs driven by mitochondrial dysfunction in the development of HO, which is mediated by KLF2‐PPARγ signalling (Figure 10). Our study provides new information on potential therapeutic strategies for trauma‐induced HO and other mitochondrial dysfunction‐related diseases.

KLF2 contains amino‐acid sequences similar to Drosophila embryonic pattern regulator Krüppel, which is reported as a transcription factor for reprogramming somatic cells into induced pluripotent stem cells.^29^ KLF2 has a vital role in maintaining cells stemness with a strong differentiation capacity, enabling them to differentiate into different directions in different culture environments, such as bone regeneration in osteogenic environment.^30^ Through immunoprecipitation assay, KLF2 could physically interact with the osteogenesis‐related gene RUNX2, to promote osteogenic differentiation.^11^ In BMSCs, a previous study showed that KLF2 could repress the transcriptional activity of NF‐κB to induce osteogenic differentiation,^31^ and the same trend was observed in periodontal ligament stem cells.^12^ This evidence of participation of KLF2 in metabolic orchestration of osteogenesis prompted us to investigate its role in HO. In this study, we found that inhibition of KLF2 reduced HO formation and osteogenesis of TSPCs, which is similar to that reported in above studies. Further, in Figure 1, apart from a statistically elevated KLF2 level at 3 weeks after burn/tenotomy, the KLF expression was also upregulated at 7 days. In fact, from our previous study, stem cells began to appear at the injury site in early inflammatory stage, and gradually accumulated and increased during the chondro‐osteogenesis stage.^32^ Therefore, KLF2 already begins to stimulate osteogenic differentiation in the inflammatory stage around the first week after injury, and this effect reaches its peak at 3 weeks in the chondro‐osteogenesis stage.

Emerging evidence has suggested that PPARγ plays an important role in skeletal homeostasis. PPARγ is a master differentiation transcription factor in adipogenesis of MSCs, reflecting its lineage commitment shaping potential in stem cell biology.^33^ When PPARγ expression is reduced, adipocyte differentiation is inhibited, whereas osteoblast differentiation is enhanced. PPARγ‐mediated osteogenesis plays an important role in various diseases such as vascular calcification^34^ and postmenopausal osteoporosis.^35^ Therefore, elevation in PPARγ expression could be a powerful force for the shift in the differentiation tendency of TSPCs, thus reprograming the healing process for the formation of HO. In this study, we demonstrated that PPARγ overexpression could decrease HO volume and osteogenesis of TSPCs, as also observed in previous studies. The mechanism by which KLF2 could inhibit PPARγ expression has yet to be fully elucidated. There are two possible mechanisms, both direct and indirect. Studies found that KLF2 was able to exert an inhibitory effect on PPARγ at the promoter level.^36^, ^37^ Two‐tandem Kruèppel binding sites, 5‐CCCACCTCTCCCA‐3 (base pairs 82–93), are present in the proximal promoter region of PPARγ. Electrophoretic mobility gel shift showed that KLF2 was able to bind this sequence and directly repress the PPARγ promoter, resulting in a 70% inhibition of promoter activity. Other studies reported that KLF2 inhibited factors such as C/EBP and ADD1/SREBP‐1c, which positively regulated PPARγ expression and function.^38^, ^39^ In line with these evidence, we also showed that the effects of PPARγ on HO and osteogenic differentiation of TSPCs was reversed by KLF2, confirming their contradictory and balanced role in the regulation of HO.

Current evidence suggests that changes in mitochondrial morphology and function are critical for stem cell.^40^ Mitochondria play a key role in the redox balance of cells, as they are the primary source of ROS.^41^ The ROS is an important factor in cellular homeostasis, which is important for the proper functioning of the cell.^42^ The redox balance is mainly regulated by the electron transport chain (ETC), located in the inner mitochondrial membrane,^43^ which consists of four enzyme complexes located in the mitochondrial inner membrane.^44^ These complexes are responsible for transferring electrons from electron donors to electron acceptors. This process produces ATP, as well as ROS, which are generated when electrons escape from the ETC. It has reported that when the redox balance of the mitochondria is disturbed, ROS can damage mitochondrial DNA, proteins and lipids, leading to cell differentiation defects which further aggravating mitochondrial dysfunction.^45^ For example, it has reported that Hedgehog signalling regulates antioxidant pathway and affects ROS generation in osteogenic differentiation of TSPCs,^46^ suggesting redox balance and ROS may be partially involved in the regulation of trauma‐induced HO. Previous study has reported that PPARγ can regulate the expression of antioxidant enzymes such as SOD2^25^ and CAT,^26^ which can further influence the mitochondrial electron transport chain and convert ROS into less harmful molecules.^47^ It is also consistent with our study. Furthermore, previous studies have also shown that PPARγ influences mitochondrial redox balance and therefore its supply of cellular energy, which in turn has an impact on stem cell differentiation.^48^ Therefore, in this study, we further explored the role of mitochondrial redox balance in HO and osteogenic differentiation of TSPCs, and the effects of the KLF2/PPARγ axis on mitochondrial metabolism. The results showed that PPARγ led to enhanced presence of mitochondrial antioxidant component that damaged the redox balance in TPSCs, which, however, could readily reversed by the KLF2 overexpression. Considering our findings that HO formation could be coordinated by the KLF2/PPARγ axis, we concluded that the osteogenesis of TSPCs during the formation of HO is dependent on KLF2/PPARγ axis by regulating the mitochondrial function and ROS production through affecting redox balance.

However, we must also point out that our study presents several limitations. First, despite the lack of consensus regarding the gold standard model of trauma‐induced HO, and although the mouse burn/tenotomy model may reflect the classical pathological model, the lack of bone damage and mechanical loading often demonstrated in the actual clinical setting may overshadow the clinical interpretation and translation of the results of this study. Therefore, further validation of the study results in other models and testing in large animals with anatomical similarities to humans are required. Second, we used chemical agonists, antagonists and AAVs in the knockdown and overexpression strategies used in this study. As the target specificity of these small molecule chemicals cannot be fully confirmed the conclusions drawn from the knockout paradigm, and our findings in this study should be confirmed in KLF2 transgenic cells or mice, which would provide stronger evidence.

## 5 CONCLUSIONS

In summary, KLF2/PPARγ axis regulates the mitochondrial function and ROS production by affecting redox balance in TSPCs, thereby promoting trauma‐induced HO formation. This study also demonstrated for the first time that mitochondrial dysfunction of TSPCs promotes HO formation, which may provide a new rationale for the development of future treatments.

## AUTHOR CONTRIBUTIONS

Ziyang Sun, Hang Liu and Yuehao Hu designed and conducted the in vitro and in vivo experiments, analysed the data and wrote the manuscript. Gang Luo conducted some of the in vitro experiments, ALP, ARS, IF and WB analysis. Zhengqiang Yuan conducted some of the in vivo experiments, micro‐CT, IHC, IF and WB analysis. Weixuan Liu, MD conducted some of experiments of PCR and ELISA. Bing Tu conducted some of experiments of animal model and cell culture. Cunyi Fan, Juehong Li and Hongjiang Ruan designed and conducted the research, wrote the manuscript, directed the project and provided funding. Ziyang Sun, Hang Liu, Hongjiang Ruan, Juehong Li and Cunyi Fan have read and verified the underlying data. All authors read and approved the final version of the manuscript.

## CONFLICT OF INTEREST STATEMENT

The authors declare no conflicts of interest.

## Supporting information


**Data S1:** Supporting Information

## Data Availability

The data sets used or analysed during the current study are available from the corresponding author on reasonable request.

## References

[1] Kazezian Z , Bull AMJ . A review of the biomarkers and in vivo models for the diagnosis and treatment of heterotopic ossification following blast and trauma‐induced injuries. Bone. 2021;143:115765. doi:10.1016/j.bone.2020.115765 33285256

[2] Joice M , Vasileiadis GI , Amanatullah DF . Non‐steroidal anti‐inflammatory drugs for heterotopic ossification prophylaxis after total hip arthroplasty: a systematic review and meta‐analysis. Bone Joint J. 2018;100‐b(7):915‐922. doi:10.1302/0301-620x.100b7.Bjj-2017-1467.R1 29954195

[3] Milakovic M , Popovic M , Raman S , Tsao M , Lam H , Chow E . Radiotherapy for the prophylaxis of heterotopic ossification: a systematic review and meta‐analysis of randomized controlled trials. Radiother Oncol. 2015;116(1):4‐9. doi:10.1016/j.radonc.2015.05.022 26163090

[4] Foruria AM , Lawrence TM , Augustin S , Morrey BF , Sanchez‐Sotelo J . Heterotopic ossification after surgery for distal humeral fractures. Bone Joint J. 2014;96‐b(12):1681‐1687. doi:10.1302/0301-620x.96b12.34091 25452373

[5] Foruria AM , Augustin S , Morrey BF , Sánchez‐Sotelo J . Heterotopic ossification after surgery for fractures and fracture‐dislocations involving the proximal aspect of the radius or ulna. J Bone Joint Surg Am. 2013;95(10):e66. doi:10.2106/jbjs.K.01533 23677367

[6] Scott‐Solomon E , Hsu YC . Neurobiology, stem cell biology, and immunology: an emerging triad for understanding tissue homeostasis and repair. Annu Rev Cell Dev Biol. 2022;38:419‐446. doi:10.1146/annurev-cellbio-120320-032429 36201298 PMC10085582

[7] Pulik Ł , Mierzejewski B , Ciemerych MA , Brzóska E , Łęgosz P . The survey of cells responsible for heterotopic ossification development in skeletal muscles‐human and mouse models. Cell. 2020;9(6):1324. doi:10.3390/cells9061324 PMC7349686 32466405

[8] Agarwal S , Loder S , Cholok D , et al. Surgical excision of heterotopic ossification leads to re‐emergence of mesenchymal stem cell populations responsible for recurrence. Stem Cells Transl Med. 2017;6(3):799‐806. doi:10.5966/sctm.2015-0365 28297577 PMC5442786

[9] Maity J , Barthels D , Sarkar J , et al. Ferutinin induces osteoblast differentiation of DPSCs via induction of KLF2 and autophagy/mitophagy. Cell Death Dis. 2022;13(5):452. doi:10.1038/s41419-022-04903-9 35552354 PMC9098908

[10] Jin H , Zhu Y , Wang XD , et al. BDNF corrects NLRP3 inflammasome‐induced pyroptosis and glucose metabolism reprogramming through KLF2/HK1 pathway in vascular endothelial cells. Cell Signal. 2021;78:109843. doi:10.1016/j.cellsig.2020.109843 33253911

[11] Hou Z , Wang Z , Tao Y , et al. KLF2 regulates osteoblast differentiation by targeting of Runx2. Lab Invest. 2019;99(2):271‐280. doi:10.1038/s41374-018-0149-x 30429507

[12] Li Z , Guo X , Wu S . Epigenetic silencing of KLF2 by long non‐coding RNA SNHG1 inhibits periodontal ligament stem cell osteogenesis differentiation. Stem Cell Res Ther. 2020;11(1):435. doi:10.1186/s13287-020-01953-8 33028420 PMC7539403

[13] Ahmadian M , Suh JM , Hah N , et al. PPARγ signaling and metabolism: the good, the bad and the future. Nat Med. 2013;19(5):557‐566. doi:10.1038/nm.3159 23652116 PMC3870016

[14] Duan P , Wang H , Yi X , Zhang H , Chen H , Pan Z . C/EBPα regulates the fate of bone marrow mesenchymal stem cells and steroid‐induced avascular necrosis of the femoral head by targeting the PPARγ signalling pathway. Stem Cell Res Ther. 2022;13(1):342. doi:10.1186/s13287-022-03027-3 35883192 PMC9327281

[15] Yuan Z , Li Q , Luo S , et al. PPARγ and Wnt signaling in adipogenic and osteogenic differentiation of mesenchymal stem cells. Curr Stem Cell Res Ther. 2016;11(3):216‐225. doi:10.2174/1574888x10666150519093429 25986621

[16] Raza SHA , Pant SD , Wani AK , et al. Krüppel‐like factors family regulation of adipogenic markers genes in bovine cattle adipogenesis. Mol Cell Probes. 2022;65:101850. doi:10.1016/j.mcp.2022.101850 35988893

[17] Luo Q , Li X , Zhong W , et al. Dicalcium silicate‐induced mitochondrial dysfunction and autophagy‐mediated macrophagic inflammation promotes osteogenic differentiation of BMSCs. Regen Biomater. 2022;9:rbab075. doi:10.1093/rb/rbab075 35480858 PMC9039510

[18] Wei Z , Guo S , Wang H , et al. Comparative proteomic analysis identifies differentially expressed proteins and reveals potential mechanisms of traumatic heterotopic ossification progression. J Orthop Translat. 2022;34:42‐59. doi:10.1016/j.jot.2022.04.003 35615641 PMC9117278

[19] Montaigne D , Butruille L , Staels B . PPAR control of metabolism and cardiovascular functions. Nat Rev Cardiol. 2021;18(12):809‐823. doi:10.1038/s41569-021-00569-6 34127848

[20] Peterson JR , Agarwal S , Brownley RC , et al. Direct mouse trauma/burn model of heterotopic ossification. J Vis Exp. 2015;(102):e52880. doi:10.3791/52880 26274052 PMC4544908

[21] Feng H , Xing W , Han Y , et al. Tendon‐derived cathepsin K‐expressing progenitor cells activate Hedgehog signaling to drive heterotopic ossification. J Clin Invest. 2020;130(12):6354‐6365. doi:10.1172/jci132518 32853181 PMC7685727

[22] Cao R , Wang G , Qian K , et al. TM4SF1 regulates apoptosis, cell cycle and ROS metabolism via the PPARγ‐SIRT1 feedback loop in human bladder cancer cells. Cancer Lett. 2018;414:278‐293. doi:10.1016/j.canlet.2017.11.015 29175458

[23] Zou Y , Watters A , Cheng N , et al. Polyunsaturated fatty acids from astrocytes activate PPARγ signaling in cancer cells to promote brain metastasis. Cancer Discov. 2019;9(12):1720‐1735. doi:10.1158/2159-8290.Cd-19-0270 31578185 PMC6891206

[24] Lee HY , Choi CS , Birkenfeld AL , et al. Targeted expression of catalase to mitochondria prevents age‐associated reductions in mitochondrial function and insulin resistance. Cell Metab. 2010;12(6):668‐674. doi:10.1016/j.cmet.2010.11.004 21109199 PMC3013349

[25] Zhao T , Lv WH , Hogstrand C , et al. Sirt3‐Sod2‐mROS‐mediated manganese triggered hepatic mitochondrial dysfunction and lipotoxicity in a freshwater teleost. Environ Sci Technol. 2022;56(12):8020‐8033. doi:10.1021/acs.est.2c00585 35653605

[26] Khoo NK , Hebbar S , Zhao W , Moore SA , Domann FE , Robbins ME . Differential activation of catalase expression and activity by PPAR agonists: implications for astrocyte protection in anti‐glioma therapy. Redox Biol. 2013;1(1):70‐79. doi:10.1016/j.redox.2012.12.006 24024139 PMC3757675

[27] García‐Macia M , Vega‐Naredo I , De Gonzalo‐Calvo D , et al. Melatonin induces neural SOD2 expression independent of the NF‐kappaB pathway and improves the mitochondrial population and function in old mice. J Pineal Res. 2011;50(1):54‐63. doi:10.1111/j.1600-079X.2010.00809.x 21062349

[28] Gao J , Feng Z , Wang X , et al. SIRT3/SOD2 maintains osteoblast differentiation and bone formation by regulating mitochondrial stress. Cell Death Differ. 2018;25(2):229‐240. doi:10.1038/cdd.2017.144 28914882 PMC5762839

[29] Nakagawa M , Koyanagi M , Tanabe K , et al. Generation of induced pluripotent stem cells without Myc from mouse and human fibroblasts. Nat Biotechnol. 2008;26(1):101‐106. doi:10.1038/nbt1374 18059259

[30] Zhou Y , Liu C , He J , Dong L , Zhu H , Zhang B , Feng X , Weng W , Cheng K , Yu M , Wang H KLF2(+) stemness maintains human mesenchymal stem cells in bone regeneration. Stem Cells Mar 2020;38(3):395–409. doi:10.1002/stem.3120 31721356

[31] Li Y , Wang J , Ma Y , et al. MicroRNA‐15b shuttled by bone marrow mesenchymal stem cell‐derived extracellular vesicles binds to WWP1 and promotes osteogenic differentiation. Arthritis Res Ther. 2020;22(1):269. doi:10.1186/s13075-020-02316-7 33198785 PMC7667798

[32] Li J , Sun Z , Luo G , et al. Quercetin attenuates trauma‐induced heterotopic ossification by tuning immune cell infiltration and related inflammatory insult. Front Immunol. 2021;12:649285. doi:10.3389/fimmu.2021.649285 34093537 PMC8173182

[33] Li Y , Jin D , Xie W , et al. PPAR‐γ and Wnt regulate the differentiation of MSCs into adipocytes and osteoblasts respectively. Curr Stem Cell Res Ther. 2018;13(3):185‐192. doi:10.2174/1574888x12666171012141908 29034841

[34] Reinhold S , Blankesteijn WM , Foulquier S . The interplay of WNT and PPARγ signaling in vascular calcification. Cell. 2020;9(12):2658. doi:10.3390/cells9122658 PMC7763279 33322009

[35] Vita F , Gangemi S , Pioggia G , Trimarchi F , Di Mauro D . Physical activity and post‐transcriptional regulation of aging decay: modulation of pathways in postmenopausal osteoporosis. Medicina. 2022;58(6):767. doi:10.3390/medicina58060767 35744030 PMC9228623

[36] Banerjee SS , Feinberg MW , Watanabe M , et al. The Krüppel‐like factor KLF2 inhibits peroxisome proliferator‐activated receptor‐gamma expression and adipogenesis. J Biol Chem. 2003;278(4):2581‐2584. doi:10.1074/jbc.M210859200 12426306

[37] Cui T , Huang J , Sun Y , et al. KLF2 inhibits chicken preadipocyte differentiation at least in part via directly repressing PPARγ transcript variant 1 expression. Front Cell Dev Biol. 2021;9:627102. doi:10.3389/fcell.2021.627102 33634127 PMC7901985

[38] Rosen ED , Walkey CJ , Puigserver P , Spiegelman BM . Transcriptional regulation of adipogenesis. Genes Dev. 2000;14(11):1293‐1307. 10837022

[39] Rosen ED , Spiegelman BM . Molecular regulation of adipogenesis. Annu Rev Cell Dev Biol. 2000;16:145‐171. doi:10.1146/annurev.cellbio.16.1.145 11031233

[40] Ludikhuize MC , Meerlo M , Gallego MP , et al. Mitochondria define intestinal stem cell differentiation downstream of a FOXO/Notch Axis. Cell Metab. 2020;32(5):889‐900.e7. doi:10.1016/j.cmet.2020.10.005 33147486

[41] Willems PH , Rossignol R , Dieteren CE , Murphy MP , Koopman WJ . Redox homeostasis and mitochondrial dynamics. Cell Metab. 2015;22(2):207‐218. doi:10.1016/j.cmet.2015.06.006 26166745

[42] Dan Dunn J , Alvarez LA , Zhang X , Soldati T . Reactive oxygen species and mitochondria: a nexus of cellular homeostasis. Redox Biol. 2015;6:472‐485. doi:10.1016/j.redox.2015.09.005 26432659 PMC4596921

[43] Scherer S . Do photosynthetic and respiratory electron transport chains share redox proteins? Trends Biochem Sci. 1990;15(12):458‐462. doi:10.1016/0968-0004(90)90296-n 1963954

[44] Vercellino I , Sazanov LA . The assembly, regulation and function of the mitochondrial respiratory chain. Nat Rev Mol Cell Biol. 2022;23(2):141‐161. doi:10.1038/s41580-021-00415-0 34621061

[45] Zhao M , Wang Y , Li L , et al. Mitochondrial ROS promote mitochondrial dysfunction and inflammation in ischemic acute kidney injury by disrupting TFAM‐mediated mtDNA maintenance. Theranostics. 2021;11(4):1845‐1863. doi:10.7150/thno.50905 33408785 PMC7778599

[46] Li G , Deng Y , Li K , et al. Hedgehog signalling contributes to trauma‐induced tendon heterotopic ossification and regulates osteogenesis through antioxidant pathway in tendon‐derived stem cells. Antioxidants. 2022;11(11):2265. doi:10.3390/antiox11112265 36421451 PMC9686894

[47] Nie S , Shi Z , Shi M , et al. PPARgamma/SOD2 protects against mitochondrial ROS‐dependent apoptosis via inhibiting ATG4D‐mediated mitophagy to promote pancreatic cancer proliferation. Front Cell Dev Biol. 2021;9:745554. doi:10.3389/fcell.2021.745554 35186942 PMC8847684

[48] Wan Y . PPARγ in bone homeostasis. Trends Endocrinol Metab. 2010;21(12):722‐728. doi:10.1016/j.tem.2010.08.006 20863714

