# Supplementary material for: KLF2/PPARγ axis contributes to trauma‐induced heterotopic ossification by regulating mitochondrial dysfunction
Source: Cell Prolif. 2023 Jun 21;57(1):e13521. doi: 10.1111/cpr.13521 (PMC10771107; doi:10.1111/cpr.13521)
Supplement: Supplementary file 1 — Data S1: Supporting Information [file CPR-57-e13521-s001.docx]

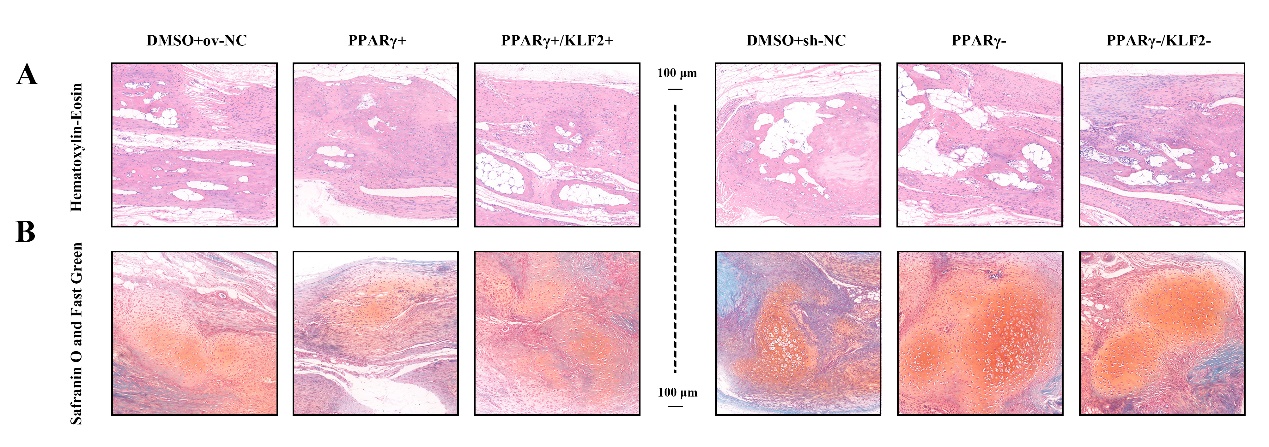


**Supplementary Figure 1 Effects of PPARγ on trauma-induced HO was regulated by KLF2**

(A) H&E staining was used to detect HO formation at 10 weeks in the tendon lesions. N = 5; scale bar = 100 μm.

(B) SOFG staining was used to detect chondrogenesis at 3 weeks in the tendon lesions. N = 5; scale bar = 100 μm.


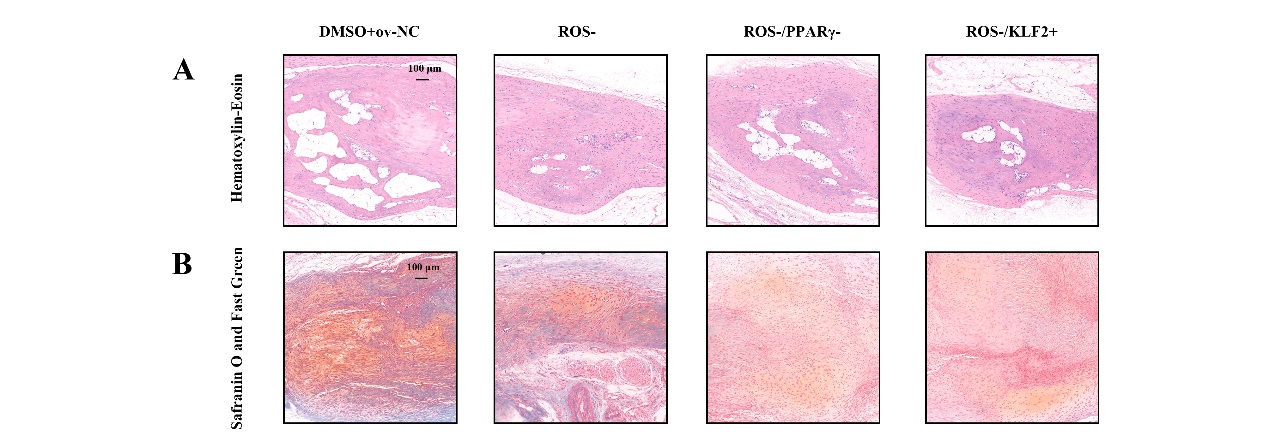


**Supplementary Figure 2 Effects of mitochondrial dysfunction on trauma-induced HO was regulated by KLF2/PPARγ pathway**

(A) H&E staining was used to detect HO formation at 10 weeks in the tendon lesions. N = 5; scale bar = 100 μm.

(B) SOFG staining was used to detect chondrogenesis at 3 weeks in the tendon lesions. N = 5; scale bar = 100 μm.


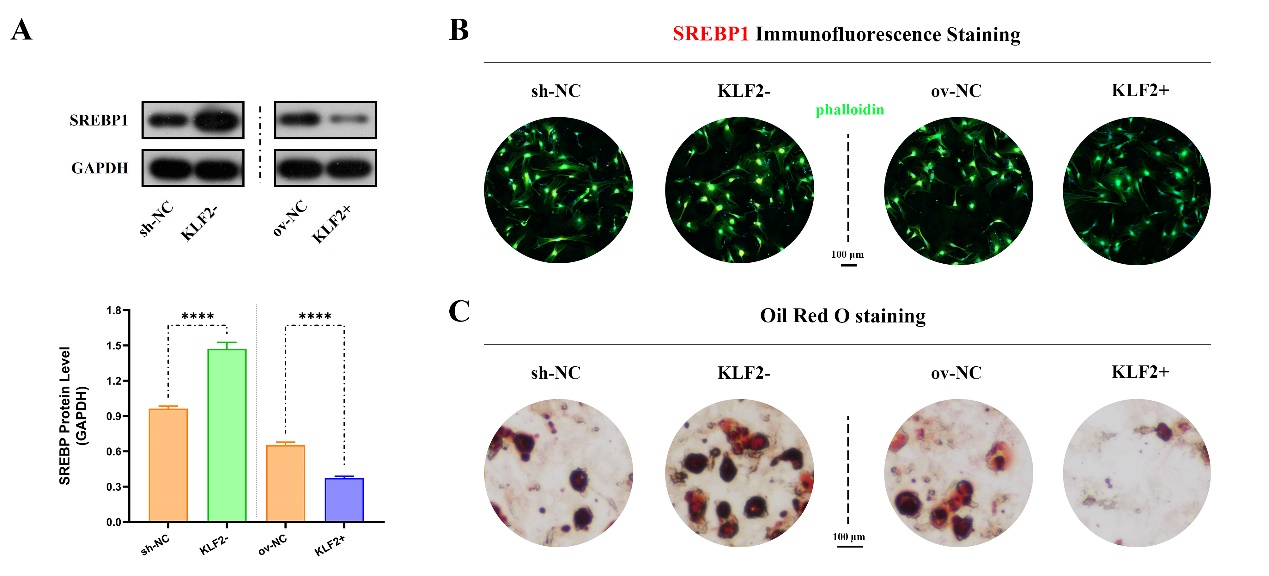


**Supplementary Figure 3 Increased adipogenesis after KLF2 inhibition in vitro for adipogenic induced TSPCs**

(A) WB analysis was used to detect the expression of adipogenesis-related proteins in the adipogenic induced TSPCs. N = 3; **** p < 0.0001.

(B) IF staining was used to detect the expression of SREBP1 (red), co-stained with phalloidin (green) and DAPI (blue), in the adipogenic induced TSPCs. N = 6, scale bar = 100 μm.

(C) Oil Red O staining was used to detect the adipogenesis of TSPCs. N = 6, scale bar = 100 μm.


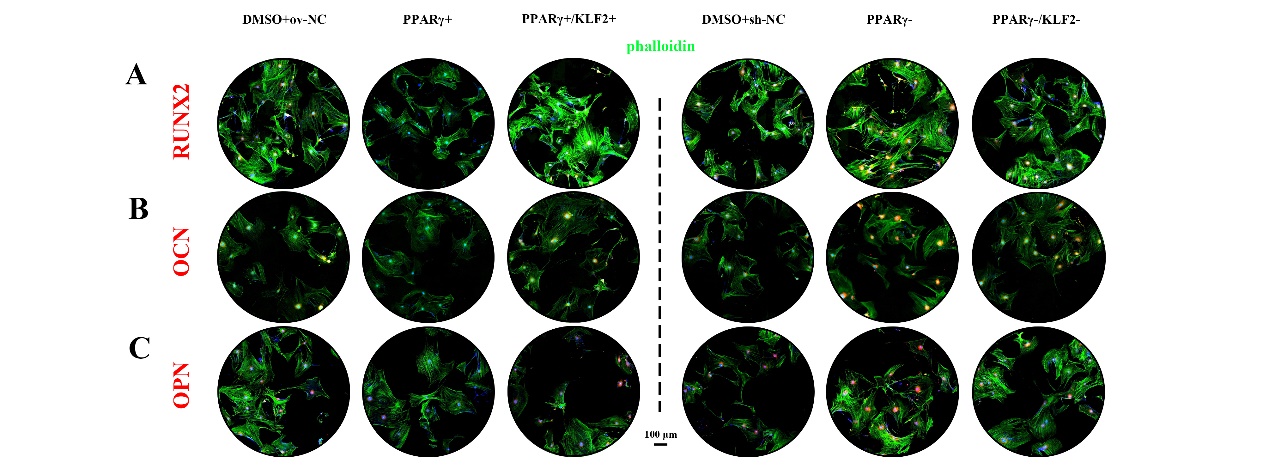


**Supplementary Figure 4 Effects of PPARγ on osteogenesis of TDSCs was regulated by KLF2**

(A-C) IF staining was used to detect the expression of RUNX2 (red), OCN (red) and OPN (red), co-stained with phalloidin (green) and DAPI (blue), in the osteogenic induced TSPCs. N = 6, scale bar = 100 μm.


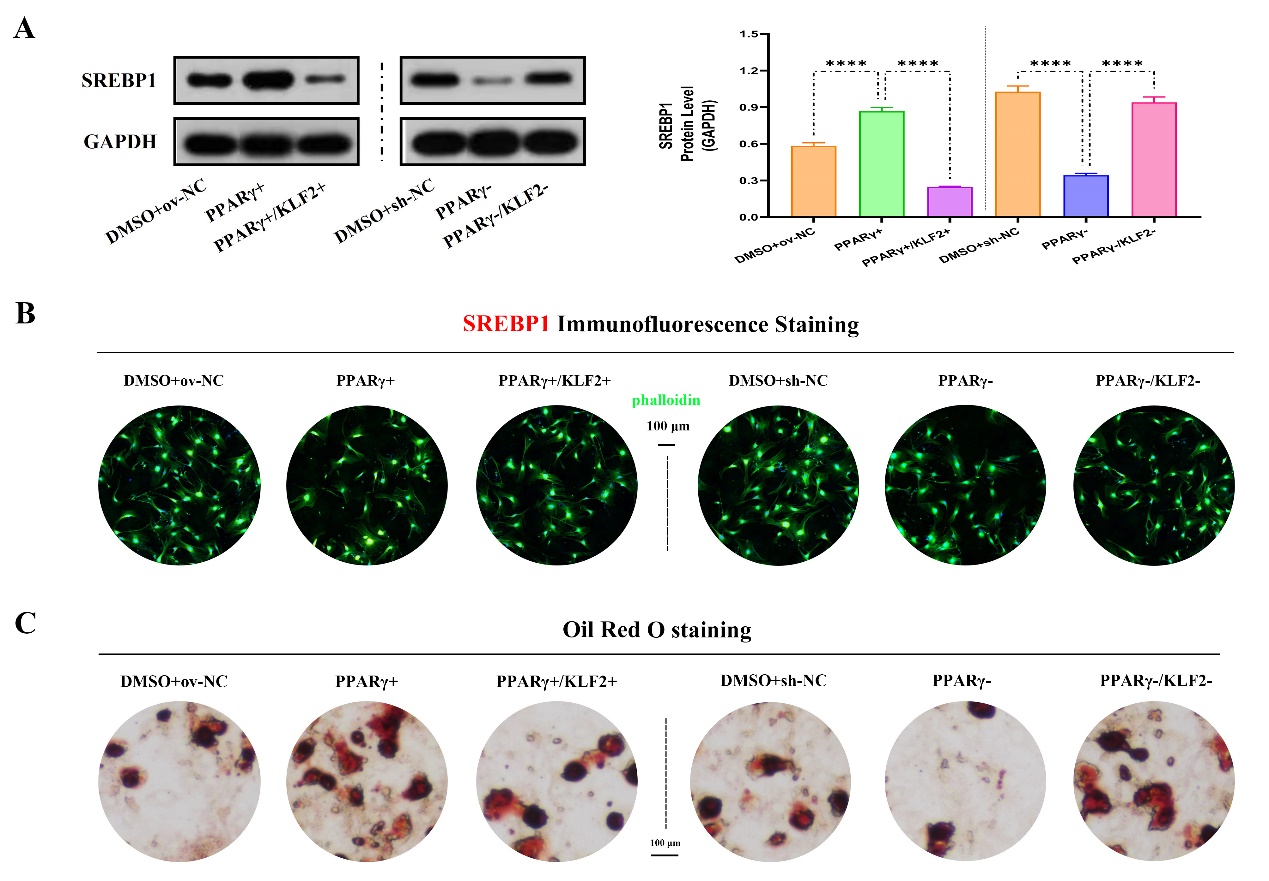


**Supplementary Figure 5 Role of PPARγ in regulation of KLF2 on adipogenesis in adipogenic induced TSPCs**

(A) WB analysis was used to detect the expression of adipogenesis-related proteins in the adipogenic induced TSPCs. N = 3; **** p < 0.0001.

(B) IF staining was used to detect the expression of SREBP1 (red), co-stained with phalloidin (green) and DAPI (blue), in the adipogenic induced TSPCs. N = 6, scale bar = 100 μm.

(C) Oil Red O staining was used to detect the adipogenesis of TSPCs. N = 6, scale bar = 100 μm.


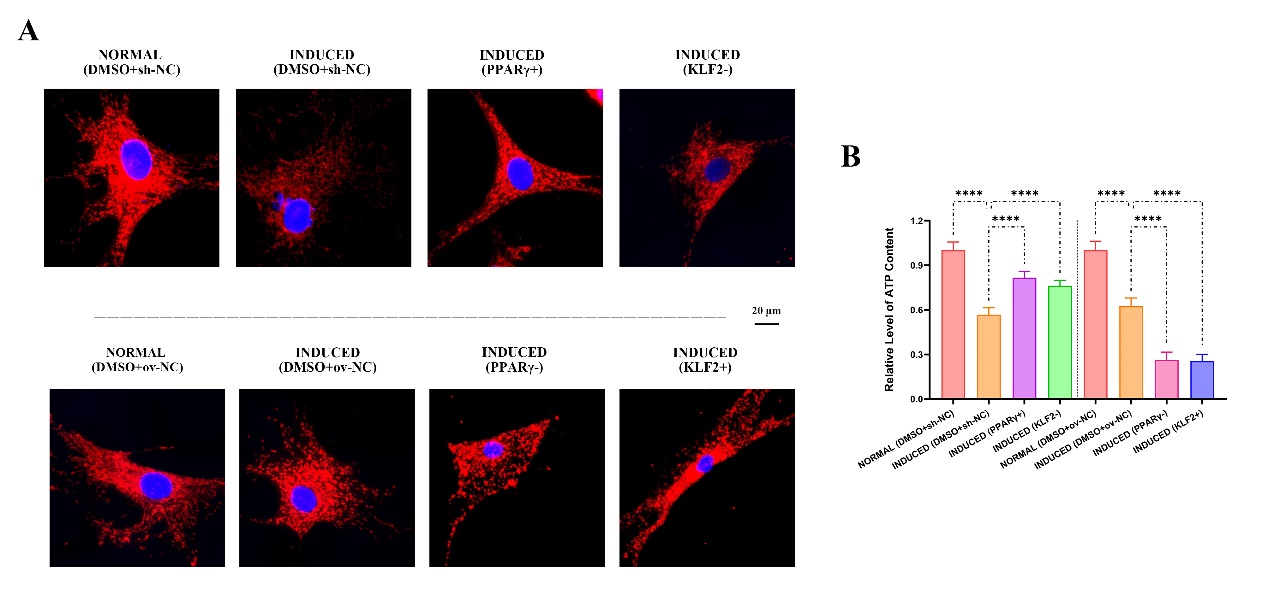


**Supplementary Figure 6 Role of mitochondrial dysfunction under KLF2/PPARγ pathway in osteogenic induced TSPCs**

(A) IF staining was used to detect the expression of TOMM20 (red), co-stained with DAPI (blue), in the osteogenic induced TSPCs. N = 6, scale bar = 20 μm. The more continuous mitochondrial network represented less mitochondrial dysfunction, while the more disrupted represented more mitochondrial dysfunction.

(B) The content of ATP level was used to detect mitochondrial function in the osteogenic induced TSPCs. N = 6; **** p < 0.0001.


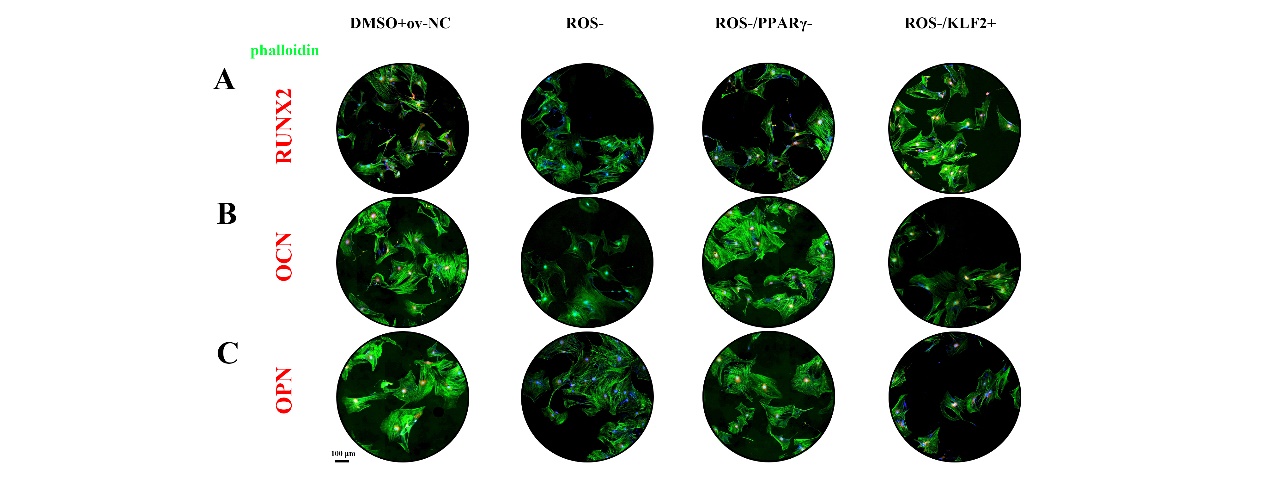


**Supplementary Figure 7 Effects of mitochondrial dysfunction on osteogenesis of TDSCs was regulated by KLF2/PPARγ pathway**

(A-C) IF staining was used to detect the expression of RUNX2 (red), OCN (red) and OPN (red), co-stained with phalloidin (green) and DAPI (blue), in the osteogenic induced TSPCs. N = 6, scale bar = 100 μm.


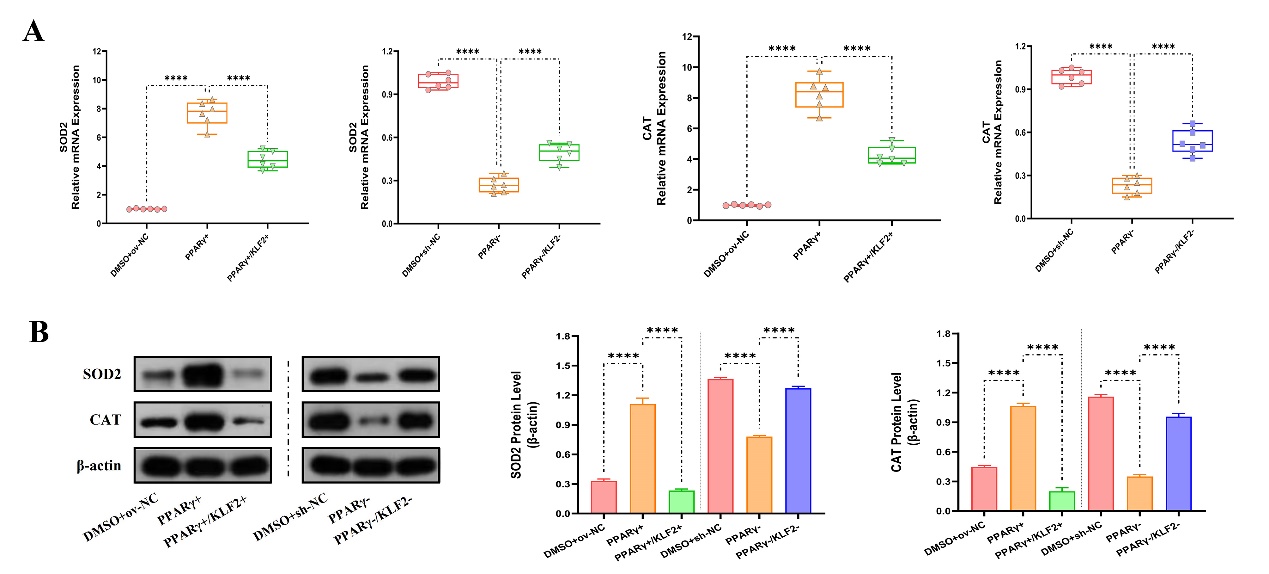


**Supplementary Figure 8 Mitochondrial redox balance regulated by KLF2/PPARγ axis in osteogenic induced TSPCs**

(A) qPCR was used to detect the relative mRNA (normalized to GAPDH) of SOD2 and CAT in the osteogenic induced TSPCs. N = 6; **** p < 0.0001.

(B) WB analysis was used to detect the expression of SOD2 and CAT in the osteogenic induced TSPCs. N = 3; **** p < 0.0001.

**Supplementary Table 1** Quantitative real-time PCR primers

| **Gene** | **Forward** | **Reverse** |
| --- | --- | --- |
| **Murine KLF2** | TTCGGTCTCTTCGACGACG | TGCGAACTCTTGGTGTAGGTC |
| **Murine PPARγ** | GCGGAAGCCCTTTGGTGACT | TGGGCGGTCTCCACTGAGAA |
| **Murine SOD2** | GCTTACTACCTTCAGTATAAA | GCCACACATTAACGCGCAGAT |
| **Murine CAT** | GAGGTCCACCCTGACTACGGG | GCCTCCTCCCTTGCCGCCAAG |
| **Murine GAPDH** | GGAGCGAGATCCCTCCAAAAT | GGCTGTTGTCATACTTCTCATGG |
